# Chitosan and Its Derivatives as Nanocarriers for Drug Delivery

**DOI:** 10.3390/molecules30061297

**Published:** 2025-03-13

**Authors:** Ranu Biswas, Sourav Mondal, Md Ahesan Ansari, Tanima Sarkar, Iustina Petra Condiuc, Gisela Trifas, Leonard Ionut Atanase

**Affiliations:** 1 Department of Pharmaceutical Technology, Jadavpur University, Kolkata 700032, WB, India; rbiswas.pharmacy@jadavpuruniversity.in (R.B.); souravtaki93@gmail.com (S.M.); mdahesanansari@gmail.com (M.A.A.); tanimasarkar19@gmail.com (T.S.); 2 Faculty of Medicine, Grigore T. Popa University of Medicine and Pharmacy, 700115 Iasi, Romania; iustina_condriuc@yahoo.com; 3 “Cristofor Simionescu” Faculty of Chemical Engineering and Environmental Protection, “Gheorghe Asachi” Technical University of Iasi, 700050 Iasi, Romania; gisela_trifas@yahoo.com; 4 Faculty of Medicine, “Apollonia” University of Iasi, 700511 Iasi, Romania; 5 Academy of Romanian Scientists, 050045 Bucharest, Romania

**Keywords:** chitosan, chemical modification, biodegradability, biocompatibility, nanoparticles, controlled release, drug delivery systems

## Abstract

Chitosan (CS) occurs naturally as an alkaline polysaccharide and has been demonstrated to have several activities of a biological nature. Additionally, as CS chains have functional hydroxyl and amino groups that are active, their applications can be expanded by chemically or molecularly altering the molecules to incorporate new functional groups. Due to its outstanding qualities, including biodegradability, biocompatibility, non-toxicity, and accessibility, it has received significant interest in all areas of biomedicine and nanomaterials being extremely promising as drug nanocarrier. The last decades have produced a lot of interest in CS-based nanoparticles (CSNPs), with an increasing number of research papers from around 1500 in 2015 to almost 5000 in 2024. The degree of crosslinking, the particulate system’s shape, size, and density, in addition to the drug’s physical and chemical properties, all have a role in how the drug is transported and released from CSNPs. When creating potential drug delivery systems based on CSNPs, all these factors must be considered. In earlier, CSNPs were employed to enhance the pharmacotherapeutics, pharmacokinetics, and solubility properties of drugs. By investigating its positively charged characteristics and changeable functional groups, CS has evolved into a versatile drug delivery system. The drug release from CSNPs will definitely be influenced by various changes to the functional groups, charges, and polymer backbone. This review mainly discusses the most important results published in the last decade. Despite the promising advantages of CSNPs, challenges related to the translation into clinical stages remain and further in vitro and in vivo studies are mandatory.

## 1. Introduction

Nowadays, nanomaterials have garnered significant interest from biomedical researchers due to their potential qualities like unique thermal, chemical, and optical capabilities [1]. These nanomaterials are utilized across a spectrum of biomedical purposes, spanning from gene transfection and transport to antimicrobial properties, drug delivery mechanisms, therapeutic systems, wound healing, and anticancer treatments [2]. Nanoparticles less than 100 nm in size performed better in terms of efficacy, site-specific drug delivery, patient compliance, and biological distribution [3]. For the purpose of enhancing the stability and bioavailability of chemically unstable bioactive substances, nanoparticles are thought to be a potential candidate [4]. They have demonstrated certain special benefits for improving the performance of several nutrients as well as drugs when used in vivo, including sustained and controlled release, longer circulation time, higher absorption properties, and fewer side effects [5]. The physically distinct, magnetic, chemical, electrical, and biological features of nanomedicine are combined with their extremely tiny size to enable easier movement inside the human body [6]. Nanomedicine has several benefits, including in vivo long-circulation, greater drug absorption, improving deep tissue permeation, and breaking down biological barriers, as well as cancer cell absorption [7]. In addition, polymeric nanoparticles (PNPs) leverage a combination of active targeting methods along with the effects of passive targeting to gain the ability to accurately locate cancer cells by identifying surface receptor expressions on tumors [8]. To achieve and maintain excellent therapeutic drug concentration at the targeted site is the main goal of drug therapy throughout the course of treatment for any condition. PNPs can regulate the gradual discharge of medications over a prolonged period, improving the therapeutic index for pharmacological action of substances [9]. Compared to standard medications, PNPs have more positive anticancer effects due to their improved absorption and fewer side effects [10].

Commercially, chitosan (CS) is typically obtained through the chitin deacetylation. Chitin is a natural linear copolymer present in various sources, like the exoskeletons of marine crustaceans (like shrimp and crabs), the outer shells of insects, and the cell walls of microorganisms [11]. It consists of β-(1–4) glycosidic bonds linking *N*-acetyl-beta-D-glucosamine and 2-amino-2-deoxy-β-D-glucopyranose units [12]. Viscometry, size exclusion chromatography, and high-performance liquid chromatography (HPLC) are frequently utilized techniques for determining the molecular weight of CS [13]. For a polymer with a semi-crystalline structure, with both strong intra-molecular and inter-molecular hydrogen bonding, CS is used in food and other products. The pharmacological use of CS is significantly influenced by its solubility. Few organic acids like formic acid, acetic acid, and lactic acid, as well as some inorganic acids like hydrochloric acid, have the ability to dissolve chitosan. Chitosan’s solubility is influenced by various factors, including its crystalline structure, deacetylation degree, molecular weight, and arrangement of acetyl groups [14,15]. CS exhibits higher solubility in acidic environments if its molecular weight is reduced [16]. Due to its chelating, antibacterial, and antioxidant activities, as well as its biopharmaceutical traits of non-toxicity, biocompatibility, biodegradability, and pH sensitivity, CS has received a great deal of interest [17,18]. CS, a cationic polymer with biological adhesion, anti-inflammatory, and cell transfection capabilities that could be improved by mixing it with other substances, has a lot of possibilities for use in biomedical applications [19]. Moreover, chitosan is capable of being cross-linked with terephthaldehyde [20], glyoxal, and glutaraldehyde to create biomaterials that may be utilized across a range of biomedical fields, consisting of gene delivery, targeted therapy as well as organ transplantation [21]. CS is utilized across different sectors, such as the pharmaceutical, cosmetic, and healthcare sectors. In the pharmaceutical sector, it is used in supplements that act to block fat and reduce cholesterol levels by removing fat and cholesterol from the body rather than permitting their absorption [22]. In the cosmetic sector, it is used in face creams, skin care products, and various other products [23], and in medical fields, it is used for wound healing and preparing tissue regeneration [24].

For the manufacturing of nanoparticles, CS is thought to be the biopolymer that is employed the most frequently due to its special characteristics [2]. The significance of CS-based nanoparticles (CSNPs) in drug delivery arises from their capacity to overcome two critical challenges often encountered with many bioactive compounds: poor aqueous solubility and low stability. These limitations hinder the effective administration and absorption of therapeutic agents, ultimately diminishing their clinical impact. Through innovative formulation techniques such as ionic gelation, emulsification, and coacervation, CSNPs effectively encapsulate hydrophilic and hydrophobic compounds, shielding them from premature degradation and enabling controlled release at specific target sites [25]. Furthermore, the tunable surface properties of CSNPs have ushered in a new era of targeted drug delivery. Techniques like PEGylation, ligand functionalization, and polymer coating afford researchers the ability to finely modulate the physicochemical attributes of nanoparticles. This makes it possible to interact with certain cellular receptors in a customized manner, facilitating site-specific delivery and reducing off-target effects, hence reducing systemic toxicity [26]. In parallel, CSNPs have made significant strides in the realm of nutraceuticals and functional foods. By encapsulating bioactive compounds like polyphenols, vitamins, and antioxidants, CSNPs confer enhanced stability, solubility, and bioavailability to these valuable substances [27]. Numerous studies have proven that CSNPs have excellent anticancer activities [28]. Applications in the biomedical field include cancer therapy, anti-HIV therapy, antibacterial action, gene transfer, drug delivery, hyperthermia therapy, wound healing, vaccine adjuvant, cell imaging, tissue engineering, and restorative dentistry [2].

Articles published in the last decade were mainly considered, ensuring the incorporation of the most relevant and up-to-date results; however, without excluding some important previously published papers.

**Figure 1:**
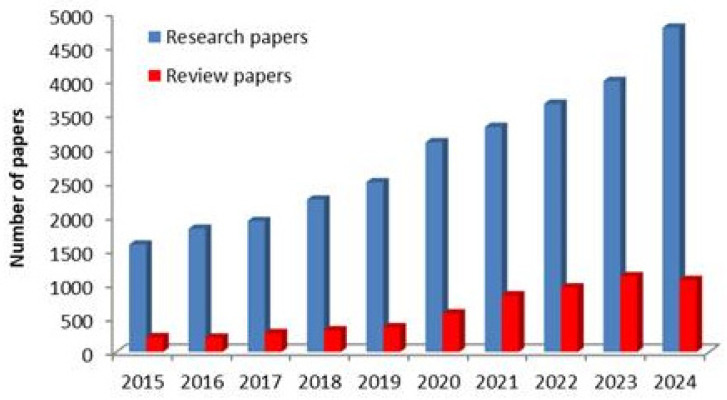
Number of published papers (research and review) per year in the last decade.

As illustrated in Figure 1, the number of papers, both research and review, published in the last decade had an increased evolution.

The search terms included combinations of keywords such as: chitosan, nanoparticles, drug delivery. Studies were screened for relevance based on their abstracts, followed by a full-text review to ensure they met the inclusion criteria.

This review will, first of all, discuss about some generalities about CS and CS derivatives, and then the preparation methods, properties and administration routes of drug-loaded CSNPs will be presented. The encapsulation of different natural active principles will be discussed before presenting some perspectives and conclusions.

## 2. Generalities

### 2.1. Sources and Structure of CS

**Figure 2:**
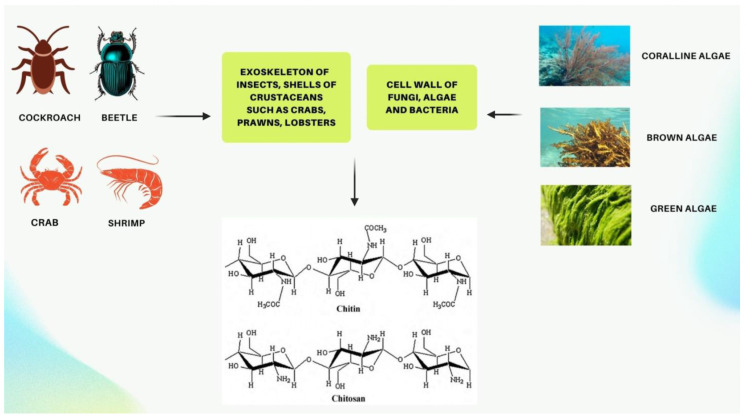
Sources and structure of chitin and chitosan [30].

CS has risen to prominence as the foremost appealing polymer in the last two decades because of its distinctive physicochemical properties and extensive range of uses in the medical field. CS is a mucopolysaccharide that occurs naturally, which resembles cellulose by chemical structure but differs in having a functional group of acetylamino [29]. In the context of commercial manufacturing, CS is produced through a process known as alkaline N-deacetylation of chitin. Chitin, a commonly found biopolymer in nature, is primarily sourced from the exoskeletons of shrimps and crabs, making it widely accessible (Figure 2) [30]. CS’s physicochemical properties are significantly influenced by the level of acetylation, whereas the deacetylation level of CS is predominantly influenced by the process of protonating glucosamine units and *N*-acetylglucosamine [31]. Depending on the origin of the chitin, its physicochemical properties are notably controlled by both its molecular weight and degree of deacetylation, including its solubility in various solvents and pH levels, hydrophobicity, and toxicity [32,33]. The glucosamine unit’s C-2 position contains an amino functional group, which significantly strengthens CS’s functional as well as structural properties. The mucoadhesiveness and cationic properties of CS resulting from the amino group make it an effective transporter for oral drug and phytochemical administration. CS is the most studied polymer in biomedical applications because of its special qualities, including biocompatibility, biodegradability, and nontoxicity as well as stability in a variety of environmental and pH conditions. The physicochemical properties of CS can be simply altered using enzymatic or chemical functionalization techniques. Modifying the amino groups and hydroxyl groups of CS yields a diverse array of customized derivatives, including *N*,*O*-modified, *N*-modified, and *O*-modified CS derivatives, which exhibit increased biological activity [28,34,35].

### 2.2. Purity of Chitosan

Chitosan purity in terms of chitin purity is very crucial as it influences the polymeric properties which may be explained as the weight % of the product excluding mainly CaCO_3_ and proteins. The purity of the extracted material obtained by the chemical or DES (deep eutectic solvents) method is listed in Table 1 [36].

### 2.3. Basic Characteristics of CS

#### 2.3.1. Different Sizes and Types of Chitosan

Chitosan biopolymer is a family of molecules having different sizes, composition, and monomeric distributions rather than a single polymer of definite structure. The number of monomeric units in the biopolymer determines the chitosan’s molecular weight (MW). Depending on the MW chitosan can be categorized into three types such as High MW chitosan, Medium MW chitosan and low MW chitosan. The physicochemical and biological properties of the polymer are highly influenced by their MW, which is summarized below [37,38] (Table 2).

#### 2.3.2. Aqueous Solubility

CS displays insolubility in water at a neutral pH, but it becomes soluble under slightly acidic conditions due to the presence of amine groups in its molecular structure. However, through quaternization, chitosan’s solubility can be enhanced in both alkaline and neutral pH environments, leading to the formation of trimethylammonium derivatives. Moreover, the molecular weight significantly influences both its solubility and degradability. Biopolymers of CS and their derivatives demonstrate improved water solubility and accelerated degradation when characterized by lower molecular weights and reduced degrees of deacetylation [39].

#### 2.3.3. Mucoadhesion

The existence of the functional group of amine imparts a cationic nature to CS biopolymers, which leads to their mucoadhesive characteristics. The adhesive effect is achieved via the establishment of hydrogen bonds among its carboxyl and amino functional groups and glycoproteins present in mucus. Mucoproteins, characterized by the existence of negatively charged molecules, attract CS with a positive charge, thereby prolonging the retention of the encapsulated drug in the gastrointestinal tract (GIT). This enhances intestinal absorption and, in turn, increases oral bioavailability. Chitosan’s mucoadhesive attributes are further heightened in environments with acidic or neutral pH levels. Greater molecular weights and levels of acetylation lead to increased mucoadhesive properties [28].

#### 2.3.4. Controlled Release

CS-based nanoparticles that contain therapeutic compounds are released through a variety of release processes, including erosion, swelling, and diffusion [31,40]. The CS-based nanoparticles experience an initial rapid release because of the swift expansion or dispersion of enclosed molecules from their outer layer. Because of the variable solubility of CS under various pH conditions, the nanoparticles also show a release of an encapsulated active principle that is dependent on the pH level. CS-based nanoparticles exhibit minimal or negligible drug release in the highly acidic environment of the stomach (pH = 1.2), while demonstrating significantly higher drug release in the alkaline environment of the small intestine (pH = 6.8). In addition, the drug release is influenced by both the molecular weight and the level of deacetylation of CS, which are further altered by its derivatization and have an important effect on the oral bioavailability of loaded active principles [41,42]. Moreover, by altering the physicochemical characteristics of CS-based nanoparticles, materials responsive to stimuli, like poly(propyl acrylic acid), have the ability to regulate the release of phytochemicals from chitosan-based nanoparticles [43]. The pH-sensitive property of CS, which involves the reversible protonation and deprotonation of its amine groups, has been widely employed to incorporate pH-responsive features [44].

#### 2.3.5. Enhancement of Intestinal Permeability

CS, a molecule which is positively charged, forms robust interactions with the mucous membrane and effectively reduces the tight junctions (TJs) between epithelial cells, thereby diminishing electrical resistance, as well as encourages passage through mucosal cells, which enhances the permeation of loaded active principles. High-molecular weight substances like phytochemicals can be delivered more effectively with the help of the chitosan’s mucoadhesive properties. As opposed to this, improved CS derivatives like trimethyl and thiolated CS significantly improve intestinal permeability. In comparison to CS, trimethyl CS has significantly greater solubility in water and greater mucoadhesion characteristics, which makes it a desirable polymer for oral administration route [39,42,45].

#### 2.3.6. Biodegradability and Safety

CS has been awarded “Generally Recognized As Safe” (GRAS) status by the Food and Drug Administration (FDA), and it has received approval for diverse applications, with a particular emphasis on its use in biomedical contexts [46]. CS’s unique characteristics, such as its mucoadhesive nature, non-toxicity, and biodegradability, make it especially suitable for use in oral drug delivery applications. CS and its derivatives, characterized by medium or low molecular weights, can be efficiently eliminated from the systemic circulation through renal clearance [47]. Enzymes and chemical reactions are primarily responsible for the degradation of CS as well as CS-based derivatives. CS with an elevated degree of deacetylation exhibits an accelerated degradation rate [48]. At present, CS is acknowledged as a safe polymer appropriate for orally delivering various bioactive substances due to its non-toxic nature [45]. Kean et al. [49] revealed that compared to compounds like sulfide (LD50 of 20 µg/mL), CS demonstrated minimal toxicity against COS7 and MCF-7 cells. Additionally, it highlighted that an increase in chitosan’s charge density could potentially raise the risk of toxicity.

Nanoparticles’ safety and toxicity profile are influenced by factors such as the polymer type, as well as size, shape, and morphology, which become crucial post-oral administration. Despite extensive research on phytochemical-loaded CS-based NPs for oral delivery in the past two decades, comprehensive in vivo evidence regarding their toxicity remains incomplete. Nevertheless, some reports based on both in vivo and in vitro models assert that CS-based nanoparticles are non-toxic and well-tolerated for oral administration [50].

### 2.4. Physicochemical Properties and Preparation of CS

**Figure 3:**
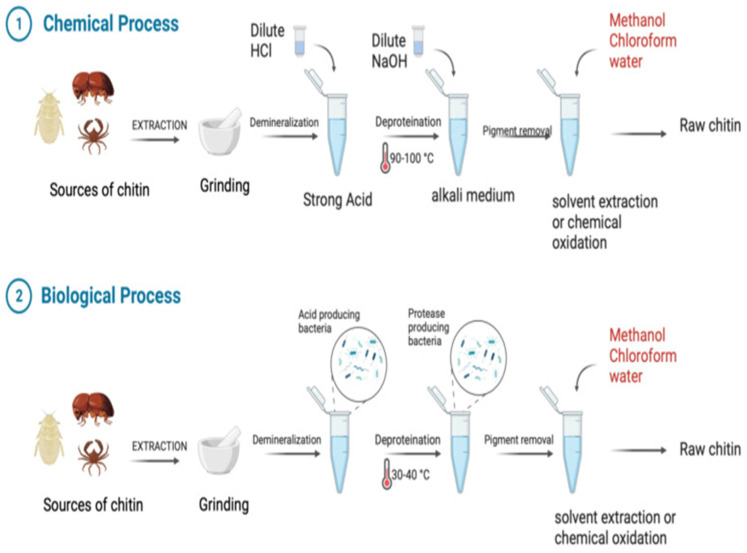
Chemical and biological processes to isolate chitin from animal sources [30].

CS is a linear binary heteropolysaccharide, comprising glucosamine units connected via β-1,4 linkages, featuring varying degrees of N-acetylation on its glucosamine leftovers [51,52]. Most commercially available CS are produced through the alkaline N-deacetylation of chitin, which is extracted from the exoskeletons of insects, the shells of crustaceans like crabs and shrimp, and fungal (aspergillus and mucor) cell walls, either by a chemical or biological process (Figure 3), and this process is relatively straightforward and accessible [30,53,54]. An alternative approach to produce CS involves enzymatic N-deacetylation, which occurs under comparatively gentle conditions [55]. From chitin, CS is created in two steps. In the first step, chitin is derived from the exoskeletons of crustaceans, which is followed by the dissolution of calcium carbonate (CaCO_3_) using a weak hydrochloric acid solution, and then the removal of proteins using a diluted aqueous sodium hydroxide (NaOH) solution. During the second phase, deacetylation of chitin occurs using 40 to 50% aqueous NaOH at temperatures ranging from 110 °C to 115 °C, conducted in an oxygen-free environment for over several hours. CS is formed when the level of deacetylation surpasses 50%. If the deacetylation of chitin becomes 75%, then also it forms CS [56]. Two essential factors which can impact chitosan’s characteristics and functionality are its level of deacetylation and molecular weight [54]. These attributes encompass characteristics, such as solubility, viscosity, coagulation reactivity with proteinaceous materials, and the chelation of heavy metal ions [57]; the moisture absorption, elasticity, elongation, and tensile strength of CS-based films are examples of their physical characteristics [58]. CS exhibits solubility in acidic water-based solutions, yet it remains insoluble in both alkaline solutions and water [52].

### 2.5. Drug Delivery Properties of CS

#### 2.5.1. Mucoadhesive Properties

The positively charged characteristic of CS is likely responsible for its mucoadhesive characteristics. In comparison to other anionic polymeric additives like hyaluronic acid, carbomer, and polycarbophil, chitosan’s mucoadhesive characteristics are inadequate [59]. A polymer must have excellent cohesive characteristics to achieve significant mucoadhesive qualities. These cohesive characteristics are frequently poor in the case of CS. Enhancement could be achieved through the creation of complexes involving polyvalent anionic pharmaceutical agents, polyvalent anionic polymeric additives, and polyvalent inorganic anions [57]. In their study, Lueßen et al. [60] showed a notable enhancement in the absorption of buserelin via the oral route in rats when co-administered with mucoadhesive polymers like carbomer. However, the desired effect could no longer be achieved when the polyanionic carbomer and CS were combined in the same formulation. The addition of trimethylated CS to PEGylated CS resulted in an enhancement of its mucoadhesive properties by up to 3.4 times [61]. Adding thiol groups to CS can greatly boost its mucoadhesive properties. Studies have shown that when in contact with the mucus gel layer, CS has the capacity to create disulfide bonds with mucus glycoproteins, rendering it the most proficient mucoadhesive polymer [62].

#### 2.5.2. Drug Delivery Properties

When a simple drug dissolution process is not sufficient to achieve the desired drug release profile, more complex techniques may be employed. One of the most used techniques is utilizing ionic interactions to regulate drug release. For cationic drugs, this method involves employing anionic polymeric excipients like carboxymethyl cellulose salt, alginate, etc., to achieve the desired effect. Nevertheless, when it comes to anionic drug delivery systems, CS stands out as the primary choice [63]. Nanoparticulate delivery systems using CS formed more stable complexes and significantly improved the absorption of drugs [64]. Mixing CS with certain anionic substances like alginate, carrageenan, polyacrylates, and pectin forms strong and stable complexes. Alternatively, CS can also be mixed with tripolyphosphate (TPP) or sulfate for similar results [59].

#### 2.5.3. Gelling Properties

When chitosan’s pH-dependent stability is appropriately managed, it can offer the benefit of in situ gelling properties as hydrogels are formed [57]. Gupta et al. [65] made a notable advancement in delivery systems that undergo gelation in situ. At pH 6.0, the researchers successfully kept their mixture of poly(acrylic acid) and CS in a liquid state but transformed into a thick gel quickly at pH 7.4. They suggested that further enhancements, such as thiolation, could improve chitosan’s in situ gelling properties. This characteristic proves valuable in areas where mucosal surfaces have access to oxygen, like the nose or eyes. When administered as a liquid with single-unit forms devoid of oxygen, a cross-linking process takes place via the creation of disulfide linkages, resulting in a significant rise in viscosity.

#### 2.5.4. Permeation Enhancing Properties

CS contains positive charges, which play a pivotal role in enhancing permeation. These charges have the potential to engage with the cell membrane, leading to a restructuring of proteins linked to or connected with tight junctions, thereby facilitating permeation enhancement [66]. The ability to enhance permeation and the level of CS toxicity were shown to be largely influenced by its structural characteristics, specifically the level of deacetylation and molecular weight [67]. CS, which has both a high molecular mass and a high level of deacetylation, showed a more substantial elevation of epithelial permeability. This phenomenon may be attributed to factors such as molecular mass, as well as the presence of other permeation-enhancing polymers like polyacrylates. CS has shown effectiveness when combined with other agents, which enhance permeation because it operates through a distinct mechanism compared to these enhancers, resulting in an additive or potentially synergistic impact [57].

#### 2.5.5. Gene Expression Properties

CS has been subject to modifications aimed at enhancing its suitability for the applications of gene expression. As an example, self-branching techniques on CS were employed to enhance their gene delivery effectiveness, and this was achieved while maintaining their safety profile [68]. Nanoparticles composed of CS and PEGylated CS have been specified as extremely promising carriers for the delivery of DNA-based therapeutics [57]. CS has the capability to create stable complexes with larger negatively charged molecules like DNA-based drugs and small interfering RNA, unlike small molecules that can only achieve controlled release of anionic drugs. When there is a sufficiently high proportion of the positively charged polymer within the complex, it results in nanoparticles with a positive charge (positive zeta potential). These positively charged nanoparticles, especially those smaller than 100 nm, can facilitate endocytosis because of their charge and size [69]. In terms of toxicology, CS is generally considered less toxic compared to other positively charged polymers like polyarginine, polyethyleneimine, and polylysine. Chitosan’s low toxicity makes it an ideal material for non-viral gene delivery. By complexing with DNA-based drugs, CS can enhance their bioavailability in the body by shielding them from degradation by enzymes called DNAses, thereby improving their delivery and efficacy [57].

### 2.6. Enhancing CS’s Properties Through Chemical Modifications

While CS offers numerous functional advantages, it also comes with certain drawbacks, including pronounced hydrophilicity, limited flexibility, significant swelling, and lower thermal stability [41]. A significant hurdle in using CS lies in its low solubility. Chitosan’s inability to dissolve at the normal pH of the body (around 7.4) limits its effectiveness as an absorption enhancer. This limitation has been a hurdle in its widespread use for biomedical applications [70]. The past decade has witnessed a surge of interest in chemical derivatives of CS due to their potential in addressing the limitations associated with unmodified CS. Notable advantages include heightened biocompatibility and improved capabilities for forming complexes with biomolecules like DNA and RNA [71]. In today’s pharmaceutical industry, the most widely utilized derivatives are produced through processes like acylation, quaternization, carboxymethylation, and thiolation [72]. In their study, Negm et al. [19] classified CS modifications into several categories: (a) substituted CS derivatives, including (i) thiolated CS, (ii) phosphorylated CS, and (iii) phtaloylated CS. (b) Cross-linked CS derivatives, such as (i) CS-glutaraldehyde cross-linked polymers, (ii) EDTA CS polymers, and (iii) CS-Epichlorohydrin cross-linked polymers. (c) Carboxylic acid CS derivatives, including (i) CS carboxyalkylate derivatives, (ii) CS methacrylate derivatives, and (iii) CS benzoylate derivatives. (d) Ionic CS derivatives, including (i) cationic CS derivatives and (ii) sulfated CS derivatives. (e) CS derivatives bound to specific molecules, such as cyclodextrin.

#### 2.6.1. Cationic and Anionic Substituents in Ionic CS Derivatives

##### Sulfated CS Derivatives

**Figure 4:**
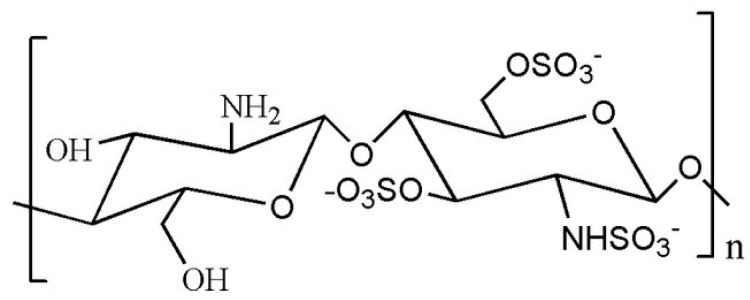
Sulfated chitosan derivatives.

CS sulfation is carried out using various sulfating agents like concentrated H_2_SO_4_, oleum, SO_3_, etc. Sulfated CS has properties like heparin, showing anticoagulant and hemagglutination inhibition effects. It also has various other benefits, such as preventing the hardening of arteries, fighting viruses, bacteria, and HIV, and acting as an antioxidant. It is useful for metal ion recovery due to its high absorption capacity. Sulfur compounds are attached to CS to collect mercury and gather valuable metals. Also, sulphonic CS works well in clumping together metal particles [73]. Ravindran et al. [73] prepared nanoparticles using curdlan sulfate and CS in a 2:1 ratio. These particles, about 205.41 ± 7.24 nanometers in size, were loaded with two active principles: Rifampicin, a powerful tuberculosis drug, and a natural compound called DdPinitol. They found that the particles with Rifampicin killed bacteria much faster (2.4 to 2.7 times) than the regular drug alone, and also reduced inflammation in cells significantly (3.66 times). Also, they noticed that when the active principles were used together, it was even more effective, reducing inflammation and boosting helpful molecule production. This suggests that these particles could be a great way to deliver different molecules into cells, especially for treating tuberculosis (Figure 4).

##### Derivatives of CS Modified with Quaternary Ammonium

**Figure 5:**
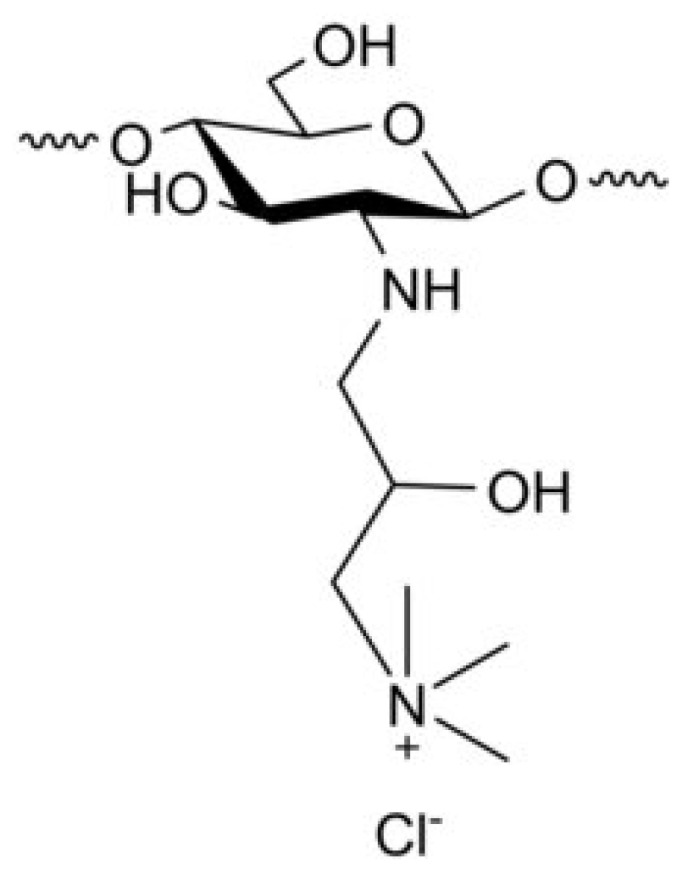
Quaternary ammonium chitosan derivatives.

The positively charged nature of CS plays a crucial role in various applications, including improving absorption, promoting adhesion to biological materials, and increasing the efficiency of introducing genetic material into cells. Additionally, it possesses properties that aid in combating cancerous growths or tumors and anti-hypercholesterolemic activities. CS derivatives with high cationic charges were synthesized through a reaction between CS and an alkyl halogenide. The procedure was performed under high-temperature conditions in an extremely basic (alkaline) environment. The highly alkaline environment was intended to counteract the acidity produced during the reaction process, thereby safeguarding any remaining primary amino groups from undergoing protonation [19]. By interacting with the anionic groups of mucins, CS acquires a more cationic character, which improves its mucoadhesive characteristics. The heightened positive charge in trimethyl chitosan (TMC), a potent cationic form of CS, leads to enhanced mucoadhesive properties and improved absorption capabilities [71]. Research indicates that a 40–60% level of quaternization is optimal for boosting absorption. Going beyond this range does not notably enhance absorption but can increase toxicity. Quaternization makes CS harder and improves its ability to remove hydroxyl radicals [72] (Figure 5).

##### Phosphorylated CS

**Figure 6:**
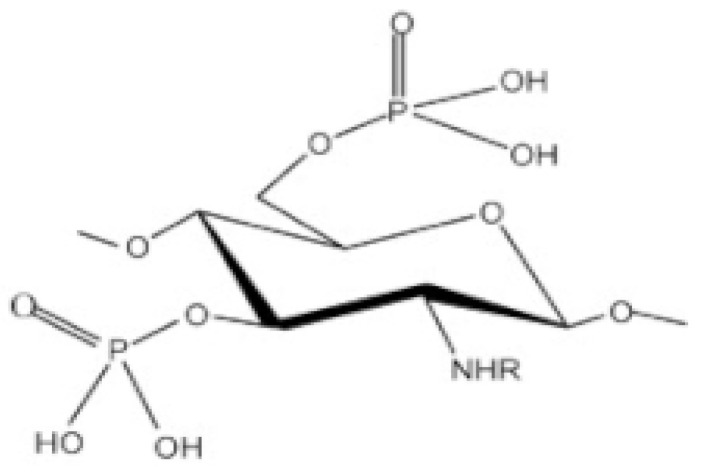
Phosphorylated chitosan.

Scientists obtained phosphorylated CS by different strategies. They used special chemicals like phosphorous pentoxide and a specific acid called methane sulphonic acid at a low temperature. Another method involved mixing CS with a kind of phosphoric acid at a high temperature, along with urea and dimethylsulfoxide (DMF). They also created it through a reaction called grafting copolymerization [70]. These modified CS derivatives were important because they could easily dissolve in water and had a talent for binding to metals. They were very useful in helping tissue regrowth and in drug delivery. Adding phosphorylcholine compounds to CS gives it anticoagulant abilities [19]. Han et al. [74] made a special material called PDLLA membrane. They then added a coating of polydopamine to the surface. After that, they added different types of CS derivatives called phosphorylated CS and/or sulfonated CS to prepare different versions of the membrane. When they tested these membranes in the lab with bone cells (MC3T3-E1s), they found that the ones with CS induce the cells grow and become bone cells better compared to membranes without CS. Specifically, when they used sulfonated CS on the outside, it helped the cells grow more, but when they used phosphorylated CS, it made the cells turn into bone cells more effectively. This suggests that both phosphorylated CS and sulfonated CS could be very useful in making materials for bone repair and growth in the future (Figure 6).

##### Succinylated CS

**Figure 7:**
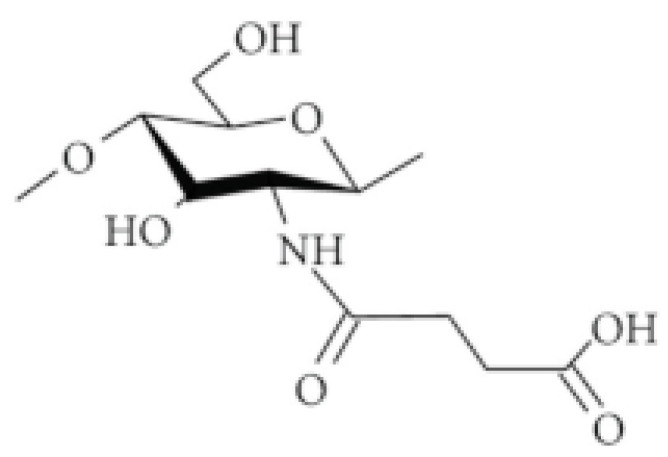
Succinylated chitosan.

N-succinyl-CS is formed via the addition of succinyl groups to the glucosamine units’ N-terminal. This process allows for easily controlling the molecular weight and the degree of substitution of the N-succinyl-CS. When there are a lot of these special groups added, it can easily dissolve in basic (alkaline) solutions, but it will not dissolve in acidic ones. This makes it significantly easier to ensure that drugs absorb better using a nasal administration route [70]. Palacio et al. [75] created nanoparticles using the succinyl-CS derivative. These particles were designed to encapsulate special compounds called polyphenols. They also conducted a theoretical study to understand how succinyl-CS and polyphenols interact with each other. The authors modified CS by adding succinyl groups through a chemical reaction and used this derivative to encapsulate three different polyphenols, epigallocatechin-3-gallate (EGCG), gallic acid (GA), and propyl gallate (PG), each with unique sizes and properties. Moreover, the encapsulation efficiency was determined, and it appeared that GA had the highest efficiency at 88%, followed by EGCG at 65%, and PG at 27%. Furthermore, a computational method, called density functional theory (DFT), was used to explore the interaction between succinyl-CS and polyphenols at the molecular level and a consistent pattern in how these molecules bind together was proposed (Figure 7).

##### Carboxyalkylated CS

**Figure 8:**
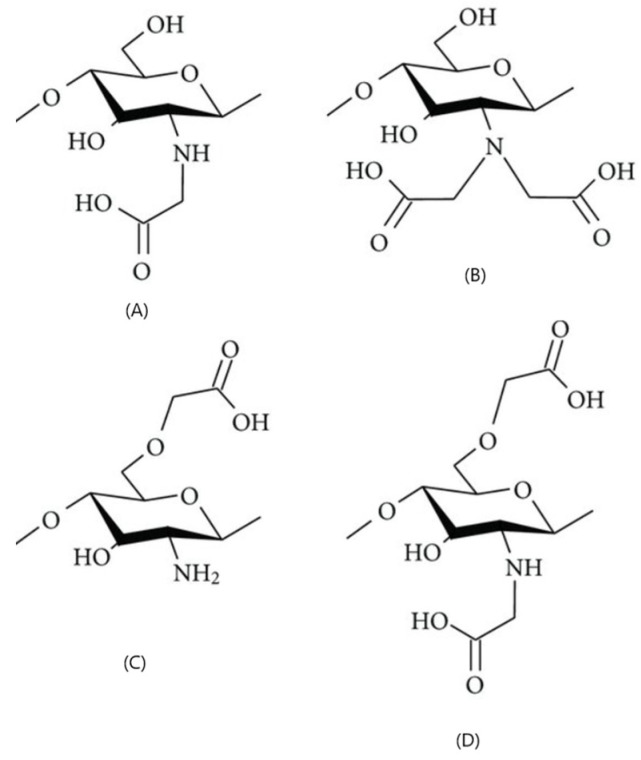
Carboxyalkylated chitosan (carboxymethylchitosan) (**A**) N-CMC, (**B**) N,N-CMC, (**C**) O-CMC, and (**D**) N,O-CMC (showing the modification at the D-glucosamine unit).

Through the process of carboxylation, acidic groups are added to the primary chain of CS, enhancing their solubility and film-forming abilities while increasing their range of applications [70]. Carboxymethyl CS is an important derivative; it dissolves in water and has properties that make it suitable for medical use. It is safe, biocompatible, breaks down naturally, helps active principles work better, and can release them in a more controlled way compared to regular CS [38]. Carboxymethyl CS has caught the attention of many different fields, including antimicrobial action, wound healing, biosensors, bio-imaging, and food production. In tissue engineering, it plays a crucial role in stopping the formation of tissue adhesions after surgery [19] (Figure 8).

#### 2.6.2. CS Derivatives Enhanced with Hydrophobic Substituents

##### Acylated CS

**Figure 9:**
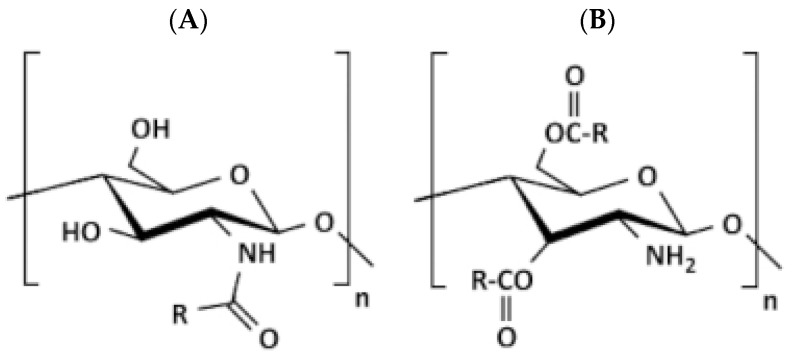
(**A**) N-acylated chitosan and (**B**) O-acylated chitosan.

Acylation is quite flexible because it allows the modification with water-repelling parts to the amino group to create an amide, the hydroxyl group to create an ester, or even both. To carry out the acylation of CS, specific derivatives of organic acid, like acid halides and anhydrides, are utilized as reagents within appropriate chemical conditions [70]. In a study, Nanda et al. [76] introduced an innovative approach involving the development of a paclitaxel-loaded liposomal formulation with a coating of acylated CS (myristoyl and octanoyl) aimed at mitigating the associated toxicities of cremophor EL. The research revealed that the liposomes with acylated CS coating exhibited a slower release of the drug compared to both CS-coated and uncoated liposomes. Moreover, liposomal formulations have shown reduced cytotoxicity compared to the direct injection of paclitaxel (marketed as Celtax™ by Celon Labs, Hyderabad, India). The results, obtained from in vitro studies evaluating liposome delivery, were achieved through the observation of cellular uptake and intracellular distribution, both with and without coating, into the cell cytoplasm. Notably, the liposomal system featuring myristoyl CS coating demonstrated superior characteristics in terms of drug absorption, distribution in the body, and targeting of tumor sites. These promising findings suggest the possible effectiveness of employing delivery systems comprising liposomes coated with acylated CS for tumor-targeting purposes (Figure 9).

##### Alkylated CS

**Figure 10:**
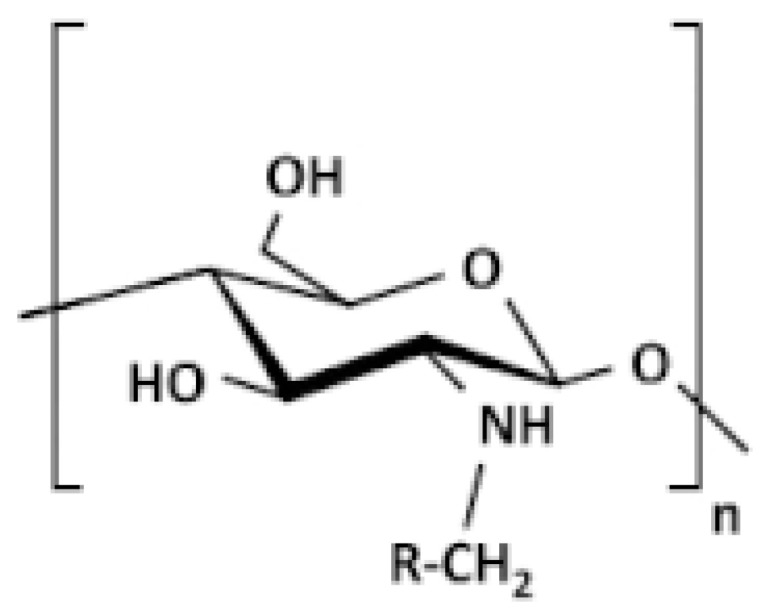
Alkylated chitosan.

Derivatives of alkylated CS are typically created through a process called reductive amination of CS. When a short alkyl chain (C5) is used, the stiffness or interaction characteristics of the modified CS are not significantly affected. However, when longer chains are used, it leads to stronger hydrophobic interactions and increased clustering of the polymer [77]. Burr et al. [78] explored two methods to modify high molecular weight CS using glycidyl trimethyl ammonium chloride (GTAC), resulting in water-soluble CS derivatives across various pH levels. Additionally, a special CS derivative was created by modifying it with a molecule called Quab 342, which has C12 alkyl chains. These derivatives can mix with anionic surfactants like SDS to make soluble complexes, and the one with Quab 342 can even create gels (Figure 10).

##### Benzoylated CS

**Figure 11:**
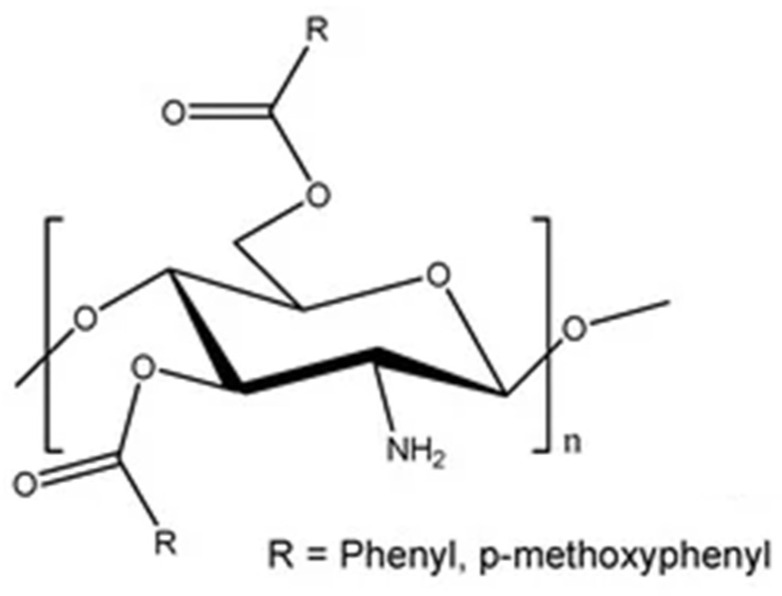
Benzoylated chitosan.

Benzoyl CS biopolymers are important in wound healing, drug delivery, cosmetics products, and chromatographic separation techniques [19]. Mohamed et al. [79] conducted a study where they chemically linked CS with different amounts of a compound called BBTUCS (4,4′-(5,5′carbonylbis(1,3-dioxoisoindoline-5,2-diyl)) dibenzoyl isothiocyanate), labeled as BBTUCS-1,2,3,4. They tested these hydrogels for their ability to inhibit COX enzymes, particularly COX-2, which is associated with inflammation. BBTU-CS-4 hydrogel stood out as a potent inhibitor of COX-2, with an IC50 value of 0.42 µg/mL, surpassing the effectiveness of the standard anti-inflammatory drug Celecoxib (IC50 0.26 µg/mL). Notably, BBTU-CS-4 showed significant promise as an anti-H. Pylori treatment, surpassing the efficacy of the other hydrogels tested (Figure 11).

##### N-Phthaloylated CS

**Figure 12:**
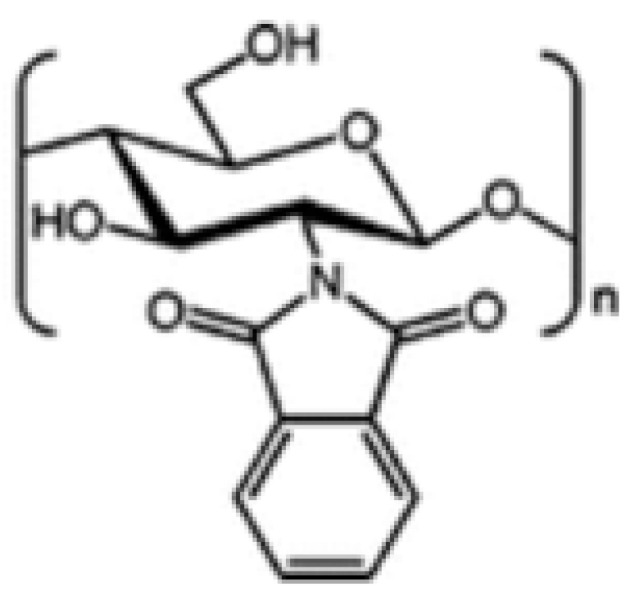
N-phthaloylated chitosan.

CS does not have good solubility in organic solvents, but when it is modified with N-phthaloylation, it becomes more soluble. When fully deacetylated, the CS polymer reacts with phthalic anhydride to create an N-phthaloyl CS derivative. This derivative can easily dissolve in solvents that have both aqueous and organic properties [19]. Permadi et al. [80] modified CS in two ways: first, they protected it with phthalic anhydride (called N-phthaloylated CS), and second, they used CS without this protection. They added certain chemical parts to the CS structure and this modified CS was suggested to make drugs dissolve better and also to increase their efficiency. It was also supposed to break down more easily (Figure 12).

#### 2.6.3. CS Derivatives with Amphiphilic Substituents

##### Cholic and Deoxycholic Acid-Modified CS

**Figure 13:**
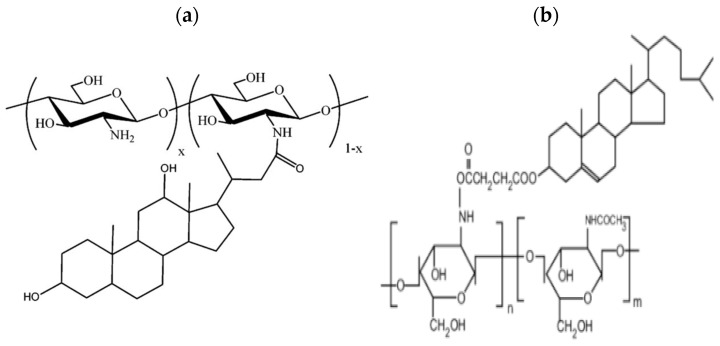
Cholic and deoxycholic acid-modified chitosan (**a**) deoxycholic acid and (**b**) cholic acid.

Cholic and deoxycholic acids have a dual nature, consisting of both hydrophilic components and a hydrophobic cyclopentanophenanthrene core. This amphiphilic character enables them to form micelle structures in aqueous environments, where the hydrophobic cores aggregate and are surrounded by the hydrophilic components, creating a stable molecular assembly in water. When cholic or deoxycholic acid is integrated into CS, it imparts self-assembling capabilities to the polymer, making it a promising candidate for transporting hydrophobic drug compounds. The hydrophobic nature of the modified CS plays an important role in safeguarding and regulating the release of the encapsulated drug [70] (Figure 13).

#### 2.6.4. CS Copolymers (with Polymer Substituents)

##### PEGylated CS

**Figure 14:**
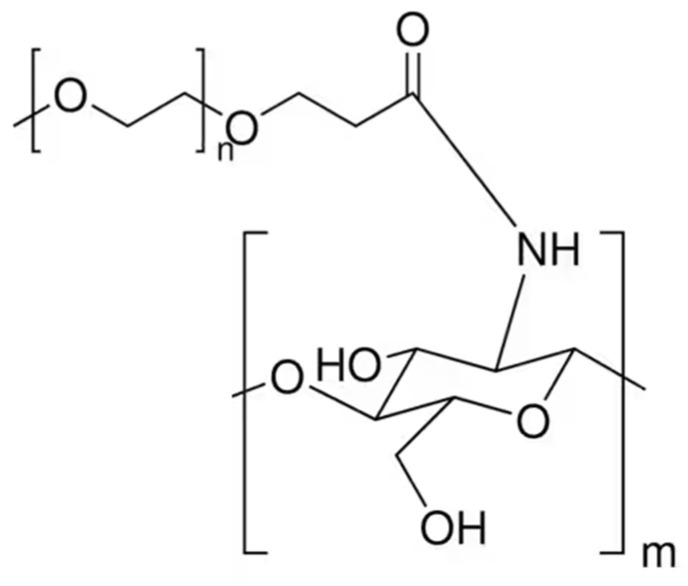
PEGylated chitosan.

By the PEGylation method it is possible to enhance the particles’ half-life which resulting in long-lasting effects, lower doses, and better patient adherence. However, PEGylated CS does not work well for mucus administration because it creates a barrier, leading to inadequate absorption. However, it proves effective for delivering genes, it shields the drug, allowing it to reach its target over an extended period [81]. Hsu et al. [82] developed self-assembled polymeric micelles using amphiphilic PEGylated CS/DBA conjugates, maximizing loading efficiency and enhancing the photostability of indocyanine green (ICG) at pH 7.4. These micelles featured a CS/DBA core and PEG corona. The ICG encapsulation was improved, but its aqueous stability also increased. The resulting micelles exhibited excellent ICG retention under acidic and physiological conditions, biocompatibility, as well as the potential for cancer theragnostic through photo-induced hyperthermia (Figure 14).

##### PEG-Methacrylate CS

**Figure 15:**
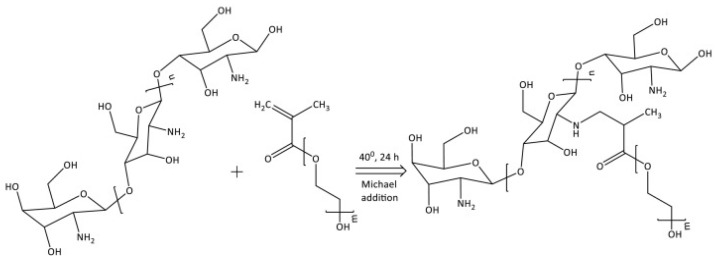
Chitosan grafted poly(ethylene glycol) methacrylate [83].

A copolymer is created by grafting poly(ethylene glycol) methacrylate onto CS through Michael addition. This double crosslinking process, involving both ionic and covalent bonds, is conducted within a reverse emulsion. This modification significantly improves chitosan’s water solubility, making it a suitable polymeric nanocarrier for drug delivery applications [83] (Figure 15).

#### 2.6.5. Sugar Bound CS Derivatives

##### Dendrimer Hybrid CS

**Figure 16:**
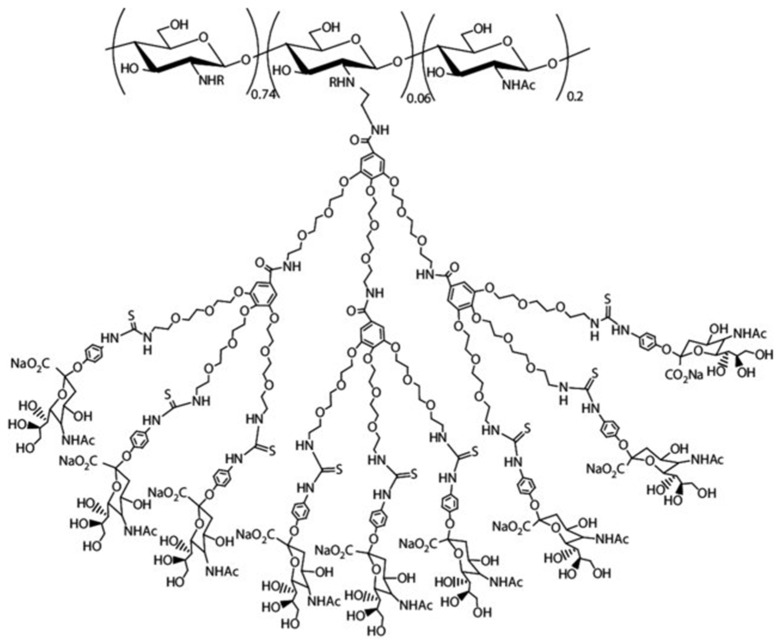
Sialo dendrimer hybrid chitosan.

Polyamidoamine (PAMAM) dendrimers are widely studied for drug delivery, especially in fields like drug transport, gene therapy, and molecular imaging. Additionally, sialo dendrimer hybrid CS structures have been researched for their potential as antiviral agents [70]. Sharma et al. [84] assessed the effectiveness of conjugated nanoparticles of CS and PAMAM, loaded with a temozolamide (TMZ) nanoformulation (CSNPs/PAMAM) in treating gliomas both in vitro and in vivo. Their research demonstrated strong cytotoxicity against T-98G and U-251glioma cell lines, and phase solubility studies of TMZ yielded exceptional outcomes. In vivo studies showed promising results, with the nanoformulation doubling TMZ concentration in the brain, highlighting the dendrimer’s surface functionality (Figure 16).

##### Galactosylated CS

**Figure 17:**
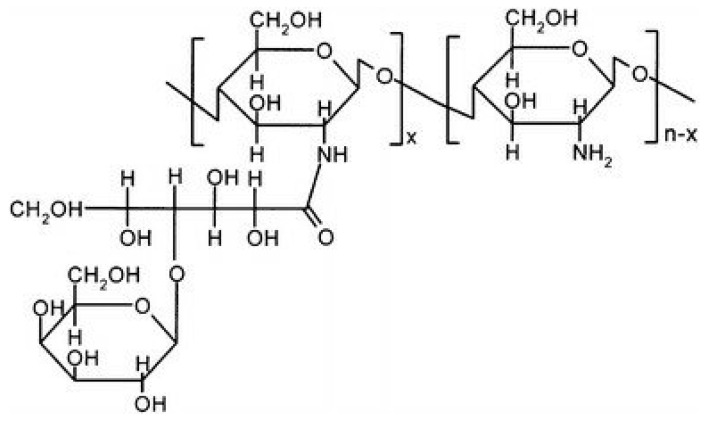
Galactosylated chitosan.

Galactosylated CS (GCS) serves as an effective carrier for targeting hepatocytes and as an artificial external cellular structure that aids in the attachment of liver cells. Moreover, GCS demonstrates promise in cancer treatment, offering increased cytotoxicity, responsiveness to pH changes, and enhanced cellular uptake [85] (Figure 17).

#### 2.6.6. Chitosan Derivatives with Cyclic Structures

##### Crown Ether-Linked CS

**Figure 18:**
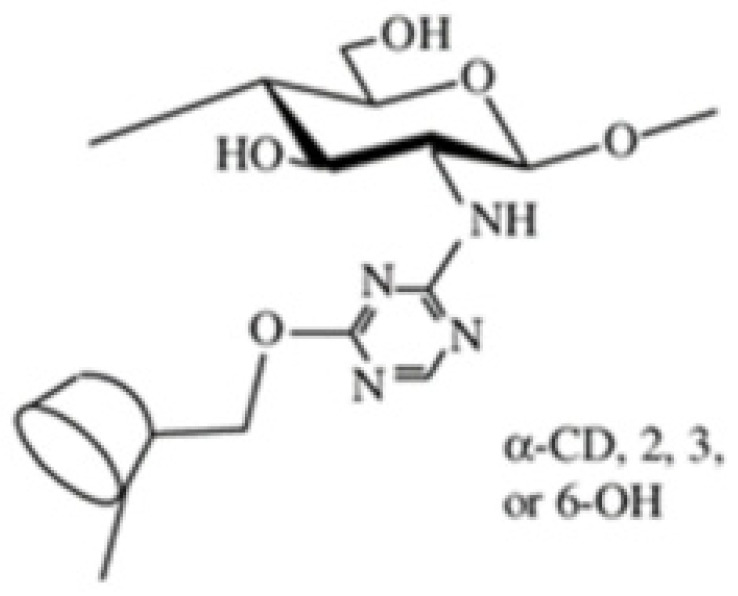
Crown ether-linked chitosan.

The composite of CS and crown ether displays an improved capacity to bind metal ions and demonstrates a greater level of selectivity for these ions, due to the combined effects of host-guest interactions and the substantial molecular weight of the CS [72]. Yi et al. [86] created a new type of CS derivative, known as CCTS-1, by combining CS with a di-crown ether compound that contains two formyl groups. This was achieved through a chemical reaction that introduced the crown ether to the CS framework. Later, they modified CCTS-1 to produce CCTS-2 by introducing secondary amine functionalities. This modification was accomplished by treating CCTS-1 with sodium borohydride, a reducing agent. CCTS-2 showed a remarkable ability to adsorb 96% of silver ions (Ag+) within just one hour at a pH of 6.0 and an initial Ag+ concentration of 0.5 mmol/L. The effectiveness of CCTS-1, CCTS-2, and their complexes with silver ions were tested against three types of bacteria. The results showed that the CCTS-2-Ag+ complex had a better ability to stop bacterial growth, compared to the CCTS-Ag+ complex, when tested against *Escherichia coli*, *Staphylococcus aureus* and *Pseudomonas aeruginosa* (Figure 18).

##### Cyclodextrin-Linked CS

**Figure 19:**
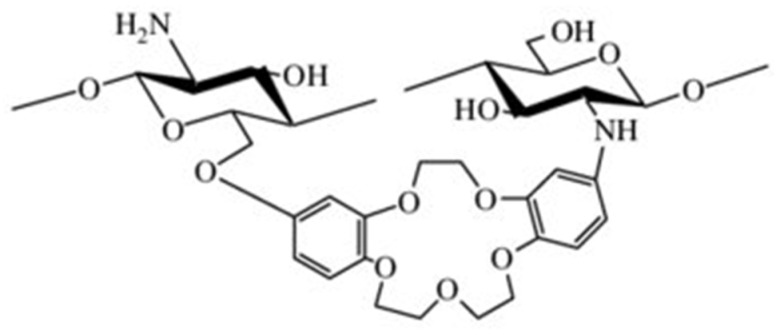
Cyclodextrin-linked chitosan.

Scientists have created a type of CS with attached cyclodextrin parts. This new material is better suited for practical applications like delivering medicine, making cosmetics, and performing chemical tests [72]. Cyclodextrin-linked CS is a special material that brings together the good qualities of CS and cyclodextrin. It can bind with different substances that have the right shape without forming strong chemical bonds [19]. Evangelista et al. [87] conducted a study where they created special complexes called supramolecular polyelectrolyte complexes (SPECs). These complexes were made using a modified form of CS with cyclodextrin attached, along with carrageenan. They tested these complexes to see how well they could release a drug called silver sulfadiazine in a controlled way. The obtained results showed that these complexes were effective against certain types of bacteria like, both Gram-positive (*Enterococcus durans/hirae* and *Staphylococcus aureus*) and Gram-negative (*Escherichia coli* and *Klebsiella pneumoniae*) (Figure 19).

#### 2.6.7. Thiol-Group Derivatives of CS

**Figure 20:**
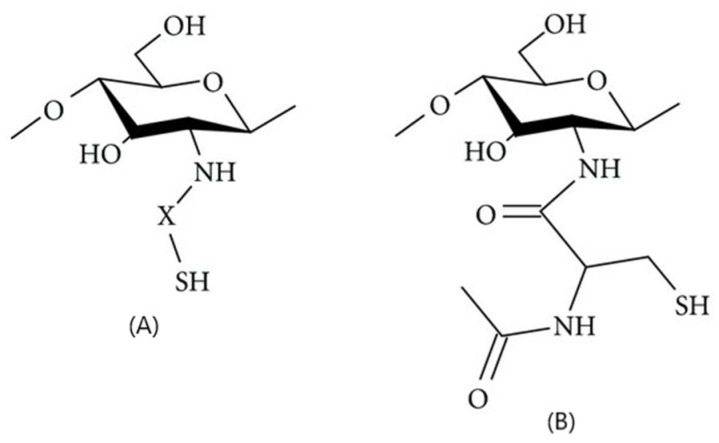
(**A**) Thiolated chitosan with the –SH group. (**B**) Thiolated chitosan with cysteine: chitosan-*N*-acetyl-cysteine.

Thiolated CS is created by attaching thiol-containing components to the two positions of glucosamine. Various thiolated CS derivatives can be produced, such as CS–thiolactic acid, CS–cysteine, CS–thioglycolic acid, CS-4–thiobutylamidine, CS–homocysteine, CS–glutathione, CS-N–acetylcysteine, CS–thioethylamidine, and CS-6–mercaptonicotinic acid conjugates [88]. Developed nanoparticles in the form of nanogels using a water-in-oil emulsion technique, by creating covalent links between thiol-modified CS (CS-SH) and a dicarboxylic derivative of poly(ethylene glycol) (PEG). The resulting amidation reaction yielded stable nanoscale networks, exhibiting positive or negative charges based on pH levels. The introduction of thiol groups significantly enhanced interactions between the nanogels and mucus. Further functionalization with a folate ligand via thiol–Michael addition (MA) showcased a convenient route for potential tumor-targeted therapies using these nanocarriers (Figure 20).

#### 2.6.8. Crosslinked CS Derivatives

##### Chitosan–Tripolyphosphate Networks

**Figure 21:**
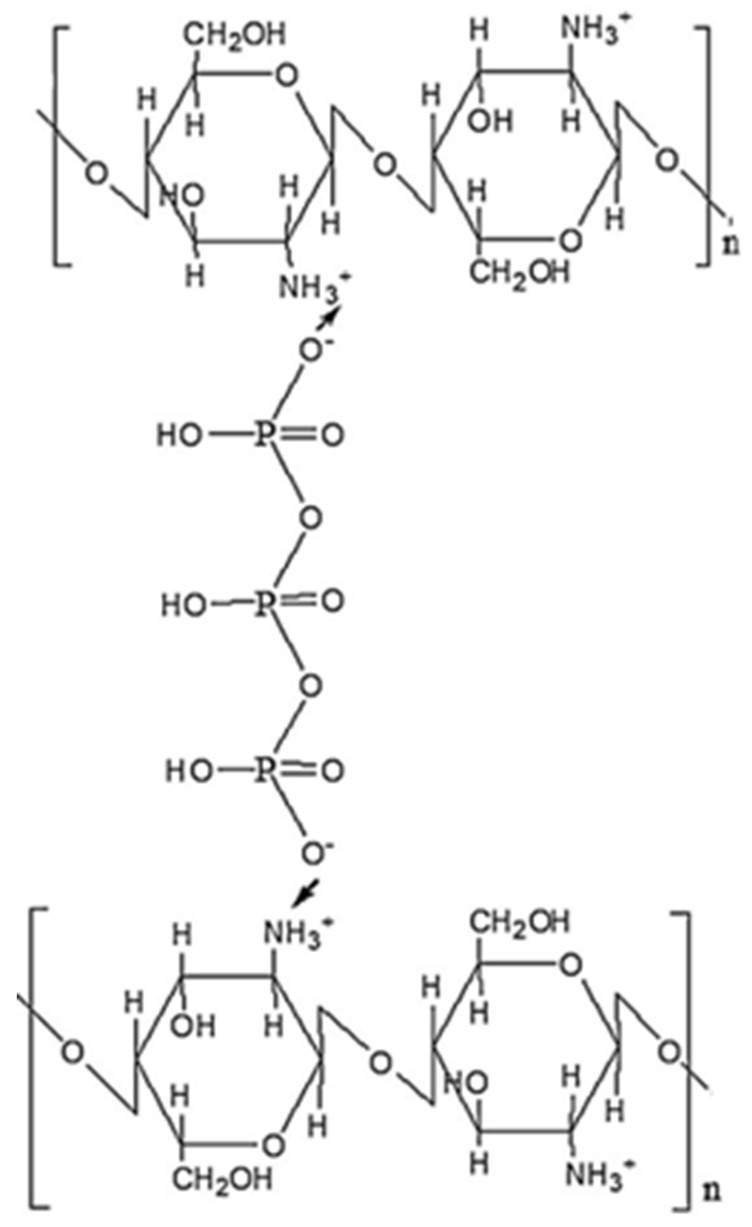
Chitosan–TPP crosslinked polymer.

Nanoparticles made from CS using ionic gelation method with TPP polymers are highly researched for drug delivery. Yet, their widespread adoption is hindered by challenges such as inconsistent results between different laboratories and a limited understanding of the physical and chemical aspects of how these particles form. These issues have been limiting their potential use in the market [89]. Sreekumar et al. [90] revealed that when maintaining a constant CS to TPP molar ratio, the average size of the resulting particles is notably influenced by the initial CS concentration. Additionally, the degree of CS acetylation emerges as the second most crucial factor in particle formation. Viscometry studies highlighted that whether salts are present or absent in the medium is a factor to consider strongly impacts both particle formation and size. By precisely modulating the initial solution concentration and its solvent conditions, it becomes feasible to consistently produce and control the attributes of CS particles, spanning from nano- to micrometers (Figure 21).

##### Chitosan–Glutaraldehyde Crosslinked Polymers

**Figure 22:**
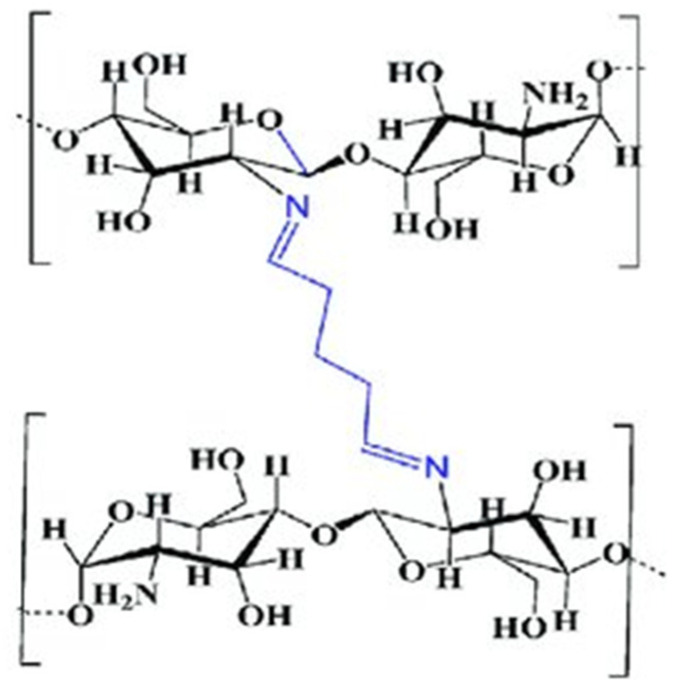
Chitosan–glutaraldehyde crosslinked polymer.

The incorporation of glutaraldehyde (GA) into CS solution resulted in improved macroscopic and microscopic characteristics, such as increased permeability, enhanced wetting properties, augmented mechanical strength, and heightened chemical resistance [70]. Yu et al. [91] developed a responsive three-dimensional hydrogel system for ophthalmic drug delivery. This system was composed of carboxymethyl chitosan (CMC) and a poloxamer consisting of a poly (ethylene oxide)/poly (propylene oxide)/poly (ethylene oxide) (PEO-PPO-PEO) block copolymer, enabling it to react to fluctuations in temperature and/or pH. The hydrogels were created by crosslinking them with GA. Rheological studies indicated that gelation occurred at 32–33 °C, with the hydrogel’s viscosity rapidly increasing post-gelation. The CCK-8 (Cell Counting Kit-8) test showed that the hydrogel and its solution are not toxic to human corneal epithelial cells when used in small amounts. Regarding drug release, nepafenac showed the highest release rate at 35 °C and pH 7.4 (Figure 22).

##### Chitosan–EDTA Conjugates

**Figure 23:**
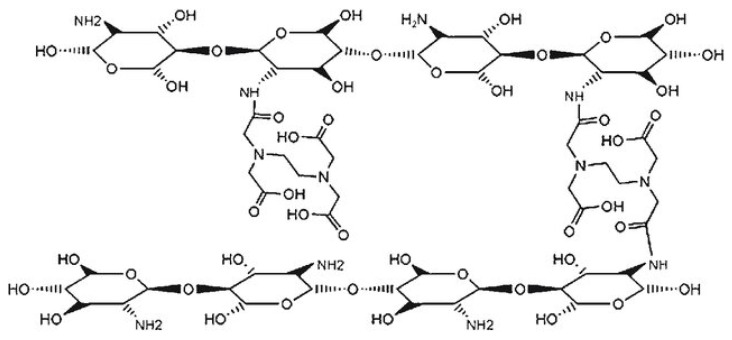
Chitosan–EDTA crosslinked polymer.

Crosslinked CS-based biosorbents play an important role in heavy metal removal, and their effectiveness is influenced by the choice of crosslinking agent and the extent of crosslinking. When CS is modified by grafting ethylene diamine tetraacetic acid (EDTA), it increases its antibacterial properties by chelating magnesium ions that in turn maintain the structural integrity of the outer membrane in Gram-negative bacteria. This modified chitosan–EDTA biopolymer also serves as a transporter matrix for controlled drug release. The control over drug release is achieved through ionic crosslinking of the biopolymer with di-cationic substances like 1,8-diaminooctane or lysine. Zhuang et al. [92] proposed a novel approach to develop an economical and highly efficient adsorbent aimed at removing strontium (Sr^2+^) and cobalt (Co^2+^) nuclides. Their study investigated the adsorption behavior and mechanism. In a system consisting of multiple components, EDTA-modified chitosan (EDTA-CS) proved to be a versatile adsorbent, demonstrating efficacy across different kinds of metal ions, even when the pH is low (pH = 1.2). The affinity of adsorption was affected by the specific arrangement of EDTA fragments and remaining CS fragments, with both coordination and electrostatic interactions contributing significantly to the adsorption process (Figure 23).

#### 2.6.9. Chitosan Derivatives Based on Thiosemicarbazone

**Figure 24:**
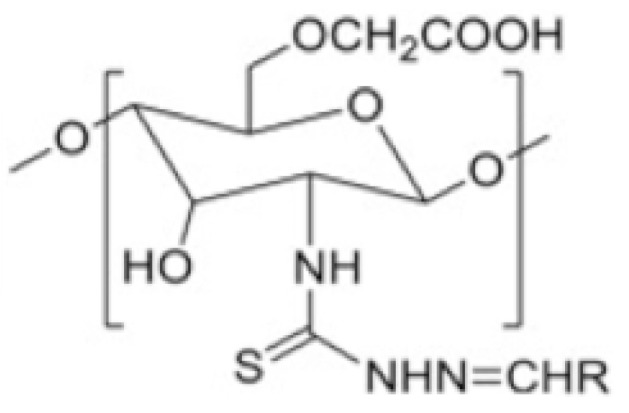
Chitosan derivatives based on thiosemicarbazone.

Diverse alterations of thiosemicarbazone can be efficiently employed to adjust the polarity and complexation characteristics of the resultant derivatives [70]. Adhikari et al. [93] have reported noteworthy advancements in thiosemicarbazone derivatives of CS, showcasing improvements in both solubility and anticancer efficacy. Their findings indicate that these derivatives possess enhanced solubility compared to the parent compound, suggesting potential advancements in drug delivery systems. Additionally, these compounds exhibited heightened anticancer properties, suggesting their potential as promising candidates in cancer therapeutics (Figure 24).

## 3. CS-Based Nanoparticles

Generally, CS-based nanoparticles (CSNPs) are employed to create stimuli-responsive carriers for chemotherapy drugs, facilitating targeted delivery to cancer sites while avoiding toxicity to regular cells. Drug encapsulation has a profound impact on the field of biomedicine as it offers a range of advantages. These include enhancing drug stability, improving distribution, increasing drug activity, and expanding bioactivity, all achieved by safeguarding pharmaceuticals against premature degradation. These measures are associated with a reduction in adverse effects. Moreover, CS-based nanoparticles demonstrate the ability to impede the growth of bacteria, exhibit antibacterial properties, and hinder bacterial ingestion in the digestive system [94]. Numerous studies indicate that CSNPs and their derivatives possess a wide-ranging effectiveness in various biomedical applications. They enhance the bioavailability, stability, and solubility of hydrophobic drugs. Additionally, they are employed for diagnostic purposes, controlled release, and as a site-specific drug delivery system [6]. Several examples will be discussed in the following sections:

### 3.1. Categorizing NPs Based on Their Structural Characteristics

Nanoparticles are typically categorized into two structural groups: nanospheres and nanocapsules. Nanospheres exhibit a uniform, solid matrix where resources are evenly dispersed. In contrast, nanocapsules adopt a classic structure of an empty shell, comprising a polymeric membrane encasing a drug-containing core. Both serve as primary candidates for incorporating active compounds, offering high payload capacity and controlled release capabilities [95]. Nano-drugs outperform current drugs and micro-structured drug combinations. They excel in precise targeting, controlled release, enhanced absorption, bioavailability, and therapeutic factor stability. Key factors influencing these capabilities include NPs’ modification, size, hydrophobicity, surface charge, and high surface-to-volume ratio. These characteristics also play a pivotal role in minimizing drug dosage as well as frequency, consequently reducing toxicity as well as potential adverse reactions associated with chemotherapy [28].

### 3.2. Preparation Methods of CS-Based Nanoparticles

CSNPs are typically employed as a transport system for a wide range of drug delivery applications. The following section provides an in-depth examination of the preparation method for CSNPs.

#### 3.2.1. Emulsion Droplet Coalescence

**Figure 25:**
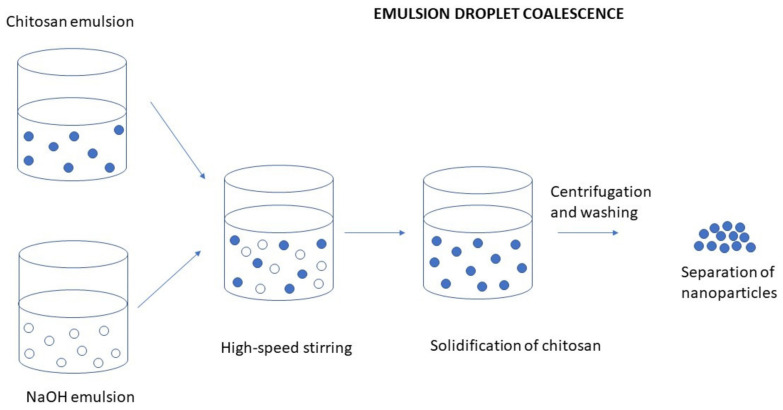
Emulsion droplet coalescence method [70].

This method involves the creation of a stable water-in-oil (W/O) emulsion by combining a liquid solution of CS and the drug with liquid paraffin, along with a stabilizer like Span 80, using high-speed homogenization. Subsequently, a second stable W/O emulsion is formed by introducing sodium hydroxide to the liquid paraffin containing the stabilizer. While blending the two emulsions, droplets from each emulsion collide and merge randomly, leading to the precipitation of CS droplets and the formation of nanoparticles (NPs) [96] (Figure 25).

#### 3.2.2. Ionic Gelation/Polyelectrolyte Complexation

**Figure 26:**
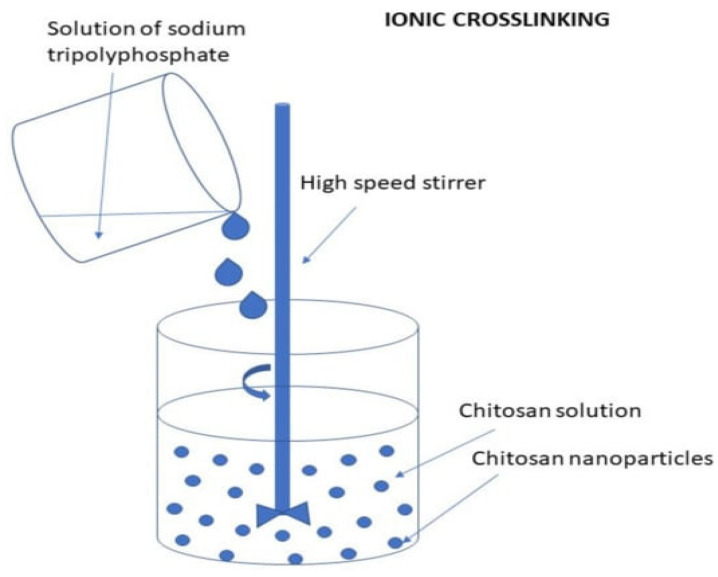
Ionic gelation method [70].

The process begins by preparing the cationic form of CS solution, achieved by introducing CS into an acidic aqueous solution. Following this, the TPP solution, in its anionic state, is added to the CS solution. The attraction between the negatively charged TPP and the positively charged CS, driven by electrostatic interactions, initiates the ionic gelation process of CS. This interaction culminates in the formation of spherical nanoparticles (NPs) with sizes under 200 nm, which were further co-loaded with trans-cinnamaldehyde (TCIN), and either curcumin (CUR) or paclitaxel (PTX) [97] (Figure 26).

#### 3.2.3. Diffusion of Solvent in Emulsion Systems

**Figure 27:**
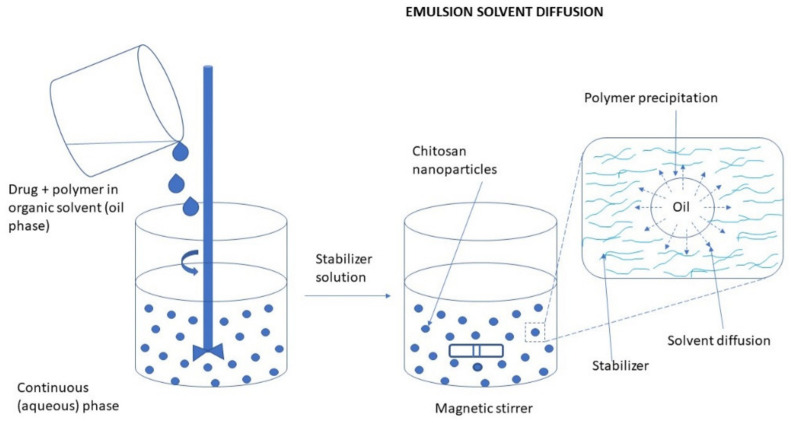
Emulsion solvent diffusion method [70].

This procedure begins with the creation of an oil-in-water emulsion, combining a hydrophilic drug with an organic phase like methylene chloride or acetone and an aqueous solution containing a stabilizer such as poloxamer or lecithin. Continuous stirring is applied during this stage. Next, high-pressure homogenization is used to eliminate methylene chloride via evaporation from the O/W emulsion. Simultaneously, the hydrophilic drug’s solubility in the aqueous phase decreases due to acetone diffusion, resulting in polymer precipitation and nanoparticle formation. To enhance acetone diffusion, additional water is often introduced. Finally, centrifugation is employed to separate the nanoparticles from the solution [98] (Figure 27).

#### 3.2.4. Desolvation

This approach begins by preparing an aqueous solution of CS. Subsequently, a precipitating substance like sodium sulfate or acetone is introduced into the CS solution, which also contains a stabilizing compound, such as Tween 80. The presence of the aqueous salty environment causes a gradual reduction in the solvation of water around the CS molecules. This reduction leads to the insolubilization of CS and the subsequent precipitation of nanoparticles. Finally, GA is introduced to solidify and strengthen the NPs [13].

#### 3.2.5. Reverse Micellization

**Figure 28:**
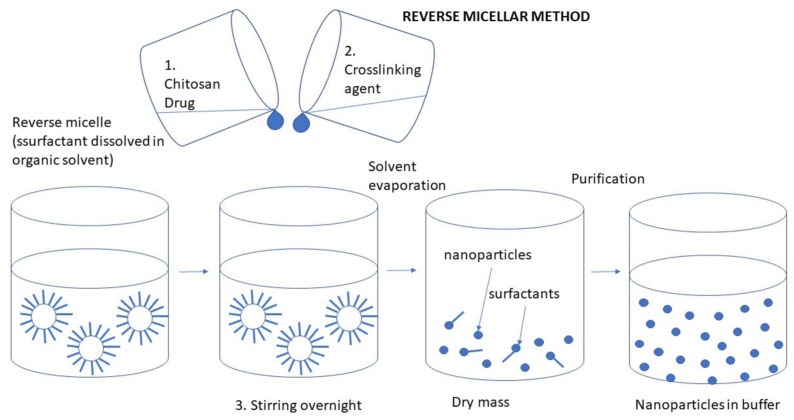
CSNPs forming by reverse micellization technique [70].

The procedure starts by preparing an organic phase utilizing a lipophilic surfactant, such as sodium bis(ethyl hexyl) sulfosuccinate or cetyltrimethyl ammonium bromide, which is then dissolved in an appropriate organic solvent like n-hexane. First, an organic phase is formed, which creates a water-in-oil (W/O) emulsion. Next, an aqueous phase is added, comprising a CS solution, the target drug, and GA. To ensure a clear mixture, the addition is carefully controlled while continuously stirring the solution to prevent any sedimentation or turbidity from occurring. Finally, the nanoparticles are extracted from the mixture [99] (Figure 28).

#### 3.2.6. Emulsification Cross-Inking

This procedure initiates with the creation of a water-in-oil emulsion, achieved by incorporating a CS solution into the oil phase. Subsequently, a relevant surfactant, such as Span 80, is employed to provide stability to the aqueous droplets within the emulsion. Following this, GA aids in creating connections between the aldehyde part and the amino part of CS, resulting in the formation of CSNPs [100].

#### 3.2.7. Combination of Ionic Gelation and Radical Polymerization

**Figure 29:**
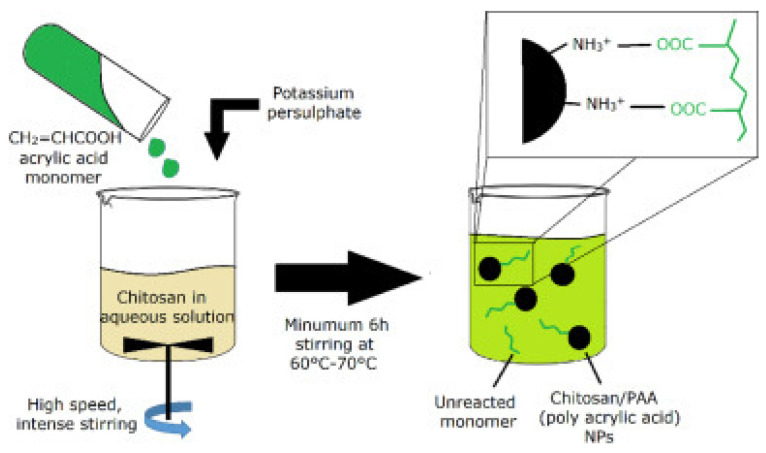
Combination of ionic gelation and radical polymerization [101].

In this process, a solution containing a monomer of acid is combined with a CS solution that can be stored at room temperature. At this point, coacervation occurs, facilitated by the communication between the cationic CS and the anionic acrylic monomer. Subsequently, the polymerization reaction is triggered between acrylic acid monomers and potassium persulfate initiator in the presence of a nitrogen stream at temperatures ranging from 60 °C to 70 °C. Following this step, the suspension of NPs is permitted to rest undisturbed overnight. Finally, any unreacted monomer is eliminated through a dialysis process [101] (Figure 29).

#### 3.2.8. Spray-Drying

**Figure 30:**
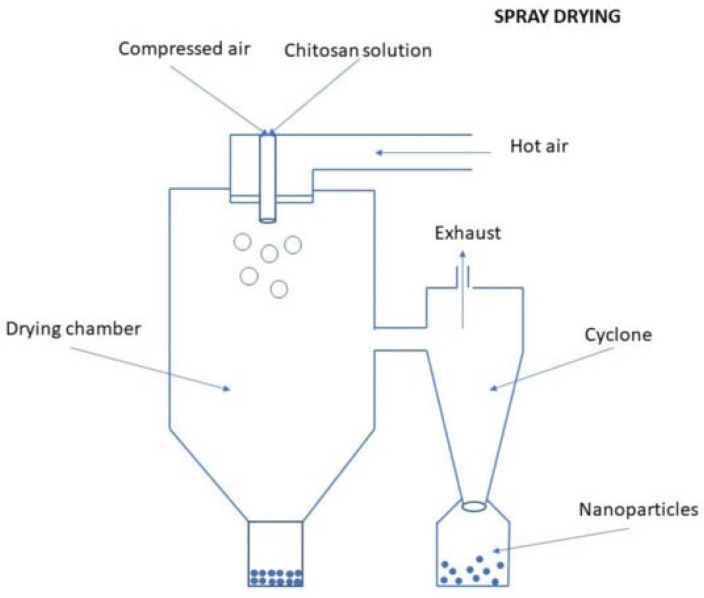
Spray-drying method [70].

In this approach, CSNPs are produced using a nano-prayed dryer. Initially, CS is dissolved in water first, and then glacial acetic acid was added and the resulting solution being stored overnight. Subsequently, the solution is atomized, transforming it into small droplets through an atomizer. These droplets are then combined with a gas that promotes evaporation to facilitate the evaporation of the liquid phase, ultimately resulting in the generation of CSNPs [13] (Figure 30).

#### 3.2.9. Nanoprecipitation

The fabrication process commences with the dissolution of CS in an appropriate solvent to form a diffusing phase. This phase is meticulously blended into the dispersing medium, methanol, via a controlled method. Utilizing a peristaltic pump set at a precise flow rate, the diffusing phase is gently infused into the dispersing phase. The injection occurs via a needle suspended approximately 2 cm above the dispersing phase’s surface, ensuring a gradual and uniform integration. Following this, Tween 80 is added to the dispersing phase, enhancing the formation of nanoparticles through its surfactant properties, thereby completing the process [102].

### 3.3. Administration Routes for CSNPs

**Figure 31:**
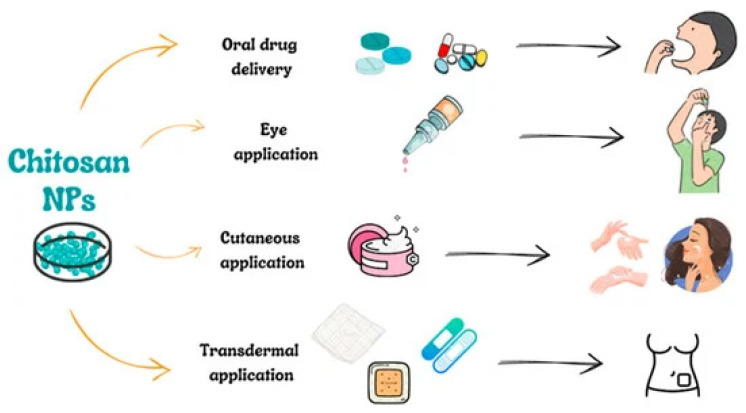
Routes of application of CSNPs [28].

CSNPs find extensive applications in biology, pharmacy, and medicine where they are highly valued for their function as conveyors for drug transport. They are commonly administered orally, topically on the skin (cutaneous), through ocular routes, and via transdermal methods (Figure 31) [28].

#### 3.3.1. Oral Administration of CSNPs

The oral administration of drugs is a mostly prevalent and straightforward approach in drug delivery systems. Its advantage lies in its non-invasive nature and patient compliance. However, it can be relatively slow, and certain medications may be vulnerable to degradation by biological fluids, like digestive acids. An optimal drug carrier would establish sturdy complexes with active substances within the gastrointestinal system, shielding them from degradation and facilitating precise delivery to cells. Additionally, the transporter must meet criteria such as being non-toxic, biodegradable, and biocompatible. CSNPs fulfill these criteria effectively [28]. Mohanbhai et al. [103] focused on formulating a treatment for targeting the powerful anti-inflammatory properties of melatonin for Crohn’s disease (CD) and ulcerative colitis (UC). The challenge lay in its limited solubility. Scientists successfully developed CSNPs that are coated with Eudragit-S-100, containing their efficacy in scavenging NO in LPS-challenged macrophages melatonin. Macrophages exposed to LPS in vitro demonstrated that melatonin effectively neutralizes nitric oxide (NO). Moreover, in vivo experiments on a UC mouse model showcased significant improvements in various pathological parameters, highlighting its potential for clinical trials.

#### 3.3.2. Ocular Administration Route of CSNPs

Hassan et al. [104] obtained a system of eye drops using CSNPs for the treatment of fungal keratitis. The optimized formulation, with particles around 200 nm in size and excellent mucoadhesive properties, demonstrated controlled drug release and safe, non-irritating characteristics in various studies. This approach holds promise for effectively treating external eye diseases, enhancing drug residence time on the cornea, and increasing bioavailability. Dealing with the difficulty of delivering medications toward the back portion of the eyeball, Yun et al. [105] developed dextran (DEX)-glycol and CS complexes, which were well-tolerated and exhibited prolonged corneal retention compared to DEX in a solution, showing potential for improved ophthalmic drug delivery.

#### 3.3.3. Cutaneous and Transdermal Administration of CSNPs

CSNPs exhibit compelling potential for biomedical use due to their distinctive characteristics. These include their capacity for cutaneous and transdermal delivery systems for both cosmetic ingredients and pharmaceutical compounds. This feature is becoming progressively significant as an alternative to overcome challenges associated with the oral administration route. In a study conducted by Ta et al. [106], it was demonstrated that CSNPs showed no toxicity to human skin fibroblasts and were able to permeate pig skin, accumulating in the dermal layer. These particles with varying molecular weights (low, medium, and high) were synthesized via ionic gelation, which was carried out utilizing two distinct cross-linking agents: Acacia and sodium TPP. The nanoparticles exhibited a spherical shape with a smooth surface and a positive charge, varying in dimensions from 200 to 300 nm, and were distributed uniformly. The research indicated that the sizes of these CSNPs were influenced by the amount of CS employed and the CS/cross-linking agent ratio. These results suggest that CSNPs could be valuable in the cosmetic sector and for delivering drugs through the skin. In another study, Tolentino et al. [107] noted an augmented targeting capability of NPs in oily conditions. They engineered NPs using CS and hyaluronic acid (Hyal), which encapsulated clindamycin for potential use as a potent system for administering therapeutic agents aimed at managing and treating acne conditions. Despite having different charges, both CS- and Hyal-based NPs exhibited similar sizes (362 ± 19 nm and 417 ± 9 nm, respectively). Notably, they showed enhanced targeted delivery of clindamycin to the pilosebaceous configuration in contrast to commercially accessible formulations that do not contain CS and Hyal. Recent literature indicates that CSNPs have demonstrated the ability to enhance anti-inflammatory and antimicrobial effects in site-specific therapy for skin-related pathogens and wound healing. CS exhibits notable antimicrobial properties, motivating researchers to develop CS-alginate NPs tailored for site-specific therapy against various pathogens, particularly addressing the pathogenesis of acne [28].

#### 3.3.4. Vaccine Delivery

CS possesses the capability to enhance the effectiveness of vaccine formulations. Administering the CS-DNA vaccine through the nasal route triggers a widespread immune reaction, leading to an elevated production of the cytokine interferon-gamma (IFN-γ) specific to the antigen. In the realm of tumor management, effective antigen displays via MHC I complexes are essential for initiating the activation of cytotoxic CD8+ T cells. CSNPs have demonstrated the ability to successfully transport and administer exogenous antigens to CD8+ cells via the MHC I complex pathway. Once taken up by dendritic cells (DCs), CSNPs are able to navigate the MHC I pathway, leading to an enhanced release of pro-inflammatory cytokines, including TNF-α, IL-1β, MCP-1, IL-6, and MIP-1α. This process also stimulates the expansion of CD4+ and CD8+ T cells, ultimately boosting the immune response [13].

#### 3.3.5. Targeting the Immune System

Both adaptive as well as natural immune responses can be elicited by CS. CS may additionally show the impact of regulated and prolonged release that can increase the amount of time that medications or antigens are present in the gastrointestinal system. CS can trigger a natural, adaptive immune response. It also has a long-lasting, controlled release that can make antigens and drugs last longer in the digestive tract. If NPs are smaller than 200 nm, they can get into the body’s lymph nodes right away after injection. But if they are bigger than 200–500 nm, antigen-presenting cells (APCs) are needed. In this case, it will take around 24 h for them to get to the nodes. In endeavors aimed at modulating or stimulating the immune response, there are two main goals. First, introducing binding molecules that interact with specific receptor proteins present on the surface of cells can target certain types of immune system cells, like DCs, and make them more likely to take up the ligands. Second, identifying certain ligands by attaching them to APCs can make vaccines even more immunogenic [108].

#### 3.3.6. Immunotherapy of Cancer

CSNPs show promise in cancer vaccine development by delivering tumor-associated antigens (TAAs) with adjuvants. They improve therapeutic outcomes by incorporating plasmids like VPIL6C, enhancing both humoral and cellular immunity. CSNPs also effectively deliver DNA vaccines against viruses like swine influenza, providing better stability and immune response compared to stand-alone vaccines. Additionally, CSNPs with various coatings demonstrate potential for drug delivery and improved bioavailability. The future direction involves optimizing CSNPs with multiple functionalities for comprehensive cancer immunotherapy [13].

### 3.4. The Protein Corona (Sometimes Called a “Protein Crown”)

The protein corona (sometimes called a “protein crown”) can form around chitosan-based nanoparticles when they are exposed to biological fluids like blood plasma or cell culture media. This phenomenon is common for many nanomaterials, including those in the chitosan particle family, such as chitosan nanoparticles (CSNPs), chitosan-coated nanoparticles, and chitosan-polysaccharide hybrid nanoparticles [109].

This protein corona is formed due to the following:
  - Surface Charge and Composition: Chitosan is positively charged (due to amine groups) at physiological pH, making it highly interactive with negatively charged plasma proteins (e.g., albumin and fibrinogen). Modified chitosan (e.g., carboxymethylated chitosan) might have different interactions based on its charge and hydrophilicity.
  - Hydrophilicity and Functional Groups: Chitosan has hydrophilic functional groups that can interact with proteins through hydrogen bonding, electrostatic forces, and van der Waals interactions.
  - Type of Surrounding Medium: In serum-containing media, chitosan particles adsorb proteins like albumin, immunoglobulins, apolipoproteins, and fibrinogen.
  - The exact protein composition depends on particle size, surface modifications, and media composition.
Implications of protein corona/crown formation:
  - Biological Fate and Circulation: The protein corona can alter cellular uptake, biodistribution, and immune response.
  - Targeting Efficiency: Some proteins in the corona can block or enhance receptor interactions, affecting drug delivery efficiency.
  - Stability and Aggregation: The corona may stabilize or destabilize chitosan particles, depending on the protein interactions.
The protein corona formation can be controlled by the following:
  - Surface Modification: PEGylation, acetylation, or grafting with other polysaccharides can reduce or modify protein adsorption.
  - Charge Tuning: Adjusting chitosan derivatives (e.g., carboxymethylation and quaternization) can influence corona composition.
  - Pre-coating Strategies: Pre-coating with specific proteins or polymers (e.g., albumin) can help control which proteins form the corona [110,111].

### 3.5. The Transcellular and Vesicular Permeability of Chitosan Nanocarrier

Chitosan nanocarriers are widely studied for drug delivery because of their ability to cross biological barriers efficiently. Their permeability depends on two major pathways:

1. Transcellular permeability (direct passage through cells) refers to the movement of chitosan nanoparticles through the cell membrane and cytoplasm before being released on the other side. Chitosan enhances transcellular transport by the following:
  - Mucoadhesive Properties: Chitosan interacts with negatively charged membrane components like sialic acid in mucins, improving retention at the absorption site.
  - Opening Tight Junctions: Chitosan transiently reversibly disrupts tight junctions in epithelial barriers (like in the intestine or BBB), allowing nanoparticles and drugs to pass.
  - Endocytosis Mechanisms: Chitosan nanoparticles are taken up via receptor-mediated, clathrin-dependent, or caveolae-mediated endocytosis, depending on their size and charge. e.g., chitosan enhances the absorption of poorly permeable drugs (e.g., insulin, heparin) via oral drug delivery, chitosan-based nanoparticles cross the blood–brain barrier (BBB) during brain delivery via receptor-mediated endocytosis, etc.
2. Vesicular permeability (transport via vesicles and endosomes) refers to the uptake of chitosan nanocarriers into endosomes, vesicles, or exosomes, allowing transport within cells before being exocytosed or degraded. Chitosan uses vesicular transport by endocytosis and intracellular trafficking (clathrin-mediated endocytosis for large or charged nanoparticles, Caveolae-mediated endocytosis for smaller, lipid-interacting particles), micropinocytosis for non-specific uptake and endosomal escape). Some chitosan formulations disrupt endosomal membranes (proton sponge effect), releasing the drug into the cytoplasm. Chitosan nanoparticles can be packed into exosomes, facilitating targeted intercellular transport, like in tumor targeting.
  Chitosan vesicular uptake is used for intracellular gene delivery (DNA/RNA transport into the cytoplasm), exosomal delivery of chitosan-based nanoparticles for drug transport into tumors, etc., are some examples of chitosan-based drug delivery. Chitosan nanoparticles use both transcellular and vesicular pathways to enhance drug permeability across biological barriers. Their ability to open tight junctions, facilitate endocytosis, and enable exosomal transport makes them highly effective for drug delivery applications [112,113].

### 3.6. The Photostability of Chitosan Nanocarrier

Light, as well as other external triggers, might alter the shape or arrangement of chitosan-based nanocarriers in plagued tissue [114]. The photostability of a chitosan-incorporated drug transport mechanism can be impacted by a number of variables, including drug nature, surface alteration, environmental pH, and degree of deacetylation (DD). For the cure of cancer, heart attacks, and neurological disorders like Parkinson’s and Alzheimer’s, the triggers might be utilized to boost cargo release throughout the affected area. NIR, or near-infrared, has the capacity to penetrate deeply into biological systems without causing detrimental adverse outcomes. For accelerating the release of the encapsulated medication, CuS’s NIR-sensitive nature makes it the perfect photothermal trigger. When adopting NIR, Mathew et al. [115] checked out the drug’s capacity to be liberated from the nanocomposite. They created covellite copper sulfide (CuS) nanospheres, implementing chitosan as a base. A conjugation between dopamine and the CuS/CS nano-drug carrier system was established. The dopamine-CuS/CS nanocomposite was put inside a dialysis bag, and the medication’s release through the carrier utilizing NIR triggering was investigated. As revealed by the cytotoxicity or cell viability experiment, the carrier and the nanocomposite are non-toxic since neither the viability of the cells nor their cell cycles were impacted. Specifically, in circumstances of neurodegenerative illnesses, this photo-controlled approach exhibited the capability to govern and regulate the targeted release of non-toxic encapsulating medications. Regarding a photo-controlled DDS on cancer therapy, Bhatta et al. [116] have documented the morphological, photophysical, and physico-chemical traits of chlorin e6 (Ce6) decorated doxorubicin (DOX) entrapped chitosan/TPP NPs created by employing an ionotropic gelation strategy. Both pH-controlled release and excellent encapsulation effectiveness towards DOX were demonstrated by the NPs. This pointed out notable anti-proliferative impacts on MCF-7 breast cancer cells following exposure to (NIR) radiation. Photo-controlled smart DOX delivery devices for cancer therapy may improve from this approach.

Substantially shielding medications from exposure to UV and visible light, chitosan nanoparticles may bring about a certain level of photoprotection, particularly when incorporated into opaque solutions. Chitosan, which originates from chitin, absorbs light mostly in the UV spectrum (200–300 nm) because of structural impurities, amino groups, and leftover aromatic molecules. It is possible to significantly enhance photostability via coating chitosan nanocarriers with UV-blocking substances such as titanium dioxide, cerium oxide, or certain flavonoids. Moreover, photostability is capable of being altered by crosslinking chitosan with agents like genipin or TPP [117,118]. Cross linkage can hinder photo-degradation in certain situations by stiffening the matrix, yet it can additionally generate photosensitive areas in other instances. Alkhader et al. reported that in comparison to free curcumin, curcumin-loaded chitosan nanoparticles heightened photostability by almost 4-times [119]. Nevertheless, extended exposure to UV light induces photo-oxidation along with the breakdown of glycosidic bonds, which lowers molecular weight and degrades polymers.

### 3.7. The Propensity of Nanoparticles to Aggregate

Nanoparticle aggregation refers to the tendency of nanoparticles (NPs) to cluster together, forming larger particle assemblies. This occurs due to interparticle interactions and can significantly impact their stability, drug delivery efficiency, and biological behavior [120].

The key factors which influence nanoparticle aggregation are as follows:

- Surface Charge (zeta potential): Nanoparticles with low surface charge (zeta potential near 0 mV) tend to aggregate due to weak repulsive forces. High positive (>+30 mV) or negative (<−30 mV) zeta potential creates strong electrostatic repulsion, preventing aggregation. Chitosan nanoparticles (positively charged) may aggregate in neutral or alkaline pH, where their charge is reduced.
- Van der Waals and Electrostatic Interactions: These forces naturally attract nanoparticles, promoting aggregation. Electrostatic repulsion (from charged surfaces) counteracts these forces. e.g., salt ions in biological fluids screen electrostatic repulsion, making aggregation more likely.
- pH and Ionic Strength of the Medium: At low pH, some nanoparticles lose their surface charge, reducing repulsion and increasing aggregation. High ionic strength (e.g., in blood or physiological fluids) reduces electrostatic repulsion, promoting aggregation. Chitosan nanoparticles aggregate in physiological pH (~7.4) due to charge reduction.
- Surface Coating and Stabilization: Hydrophilic polymers (e.g., PEGylation and polysaccharides) reduce aggregation by steric hindrance. Protein corona formation in biological fluids can sometimes stabilize or destabilize nanoparticles. E.g., PEG-coated chitosan nanoparticles resist aggregation better than uncoated ones.
- Hydrophobic vs. Hydrophilic Interactions: Hydrophobic nanoparticles (or hydrophobic regions) attract each other, leading to aggregation in aqueous environments. Hydrophilic nanoparticles (e.g., carboxylated chitosan NPs) remain better dispersed in water.

There are various consequences of nanoparticle aggregation like reduced bioavailability (large aggregates are harder to absorb across biological barriers), altered drug release (aggregation can slow or prevent drug release from nanocarriers), unpredictable biodistribution (aggregated NPs may be cleared faster or cause unintended immune responses). The aggregation can be prevented by several methods like surface modification (PEGylation, polysaccharide coatings like guar gum or pullulan), by optimizing pH and ionic strength in formulations, by adjusting zeta potential to maintain repulsive forces, by using surfactants or stabilizers to enhance colloidal stability, etc. [121].

### 3.8. Thermodynamic Perspective of Chitosan Naoparticles

The fabrication and optimization of chitosan-derived nanocarriers could potentially be anticipated based on a thermodynamic understanding of the ingredient. In order to influence the stability, release patterns, and efficacy of therapy, researchers need to manage enthalpy-entropy equilibrium at each phase (i.e., formation, loading, and release). The rational preference for chitosan derivatives, crosslinkers, and surface modifiers is further assisted by this strategy, thereby enabling customized systems to deliver drugs for a variety of therapeutic purposes [122].

The most common techniques for creating chitosan nanoparticles are emulsion-based, coacervation, and ionic gelation. Thermodynamic considerations regulate the process in each instance. The free-energy shift, ΔG < 0, which is dependent on enthalpic (ΔH) and entropic (ΔS) contributions that are increasing assemble attributes, is the cause of spontaneous formation [123]. Furthermore, chitosan stays in a molecularly dissolved form below the critical aggregation concentration (CAC); beyond CAC, thermodynamically advantageous self-assembly takes place, propelled by electrostatic and hydrophobic dynamics. Chitosan-drug compatibility (Δδ) and partition coefficient (log P) affect encapsulation effectiveness. Encapsulation is favored by closer solubility parameters (Δδ = 0), which signify greater thermodynamic compatibility. Low compatibility (high Δδ) lowers loading efficiency by raising system enthalpy [124]. Surface-free energy, protein arc formation, and phase splitting are all connected to stabilization in biological fluids throughout their storage. A greater zeta potential (ζ > ±30 mV) stabilizes the dispersion by minimizing aggregation (colloidal stability). Because of the enthalpy gain from surface attachment, plasma protein molecules adsorb towards nanoparticles in biological settings. This phenomenon impacts the effectiveness of delivery. When certain solvents are thermodynamically incompatible, precipitation or coalescence may result [125]. To balance stability with release control, intra-particle bridging improves mechanical stability by lowering ΔH swelling while limiting chain mobility by lowering entropy. Employing kinetics along with isotherm models, Ahmad et al. [126] inquired about the adsorption behavior of thiosemicarbazide chitosan (TSCs). For every concentration range, pseudo-second-order kinetics was identified through analysis of thermodynamic parameters. The estimated thermodynamic variables, encompassing ΔG, ΔH, and ΔS, remained −2.33 kJ mol^−1^, 570.40 J mol^−1^, and 9.75 J mol^−1^K^−1^ correspondingly. These findings suggested that the adsorption mechanism was physisorption and thermodynamically spontaneous.

## 4. Physicochemical Properties of CSNPs

### 4.1. Zeta Potential and Particle Size

Nanoparticle zeta potential depends on particle surface modifications and can be positive, negative, or neutral. Particle size is measured using photon correlation spectroscopy, which analyses light dispersion due to Brownian motion. The morphology and particle size of CPNPs can be characterized using transmission electron microscopy (TEM), atomic force microscopy (AFM), and scanning electron microscopy (SEM). The sizes of CPNPs typically vary between 100 and 400 nanometers [127].

### 4.2. Stability

Nanoparticle stability is crucial in determining the effectiveness of pharmaceutical products. Physical stability is affected by factors like particle agglomeration, bridging flocculation, and coagulation. On the other hand, chemical stability depends on variables like temperature, the medium’s pH, and formulation composition; it is crucial in determining the molecular weight and type of the polymer [13].

### 4.3. Cytotoxicity Study and Cellular Uptake

In assessing the cytotoxicity of CSNPs, cell viability is evaluated. This is accomplished using a straightforward, non-radioactive assay called MTT (3-(4,5-dimethylthiazol-2-yl)-2,5-diphenyltetrazolium bromide). Additionally, the efficient cellular uptake of CSNPs is studied through Confocal Microscopic Analysis (CMA) [128].

### 4.4. Drug Loading and Drug Release

Chemotherapeutic agents readily attach to nanocarriers through either covalent bonding or adsorption. There are two approaches to loading drugs onto NPs: during particle formation and post-particle formation by diffusion [28]. The active pharmaceutical ingredient can be either incorporated into the polymer matrix through physical implantation or adsorbed onto its surface. The effectiveness of this process is significantly influenced by the chosen preparation method of nanocarriers and the chemical and physical characteristics of the pharmaceutical active principle. The maximum drug loading achievable during particle formation may be affected by various factors, such as the presence of additional additives in the formulation or the purification steps [129]. CS-based particles are highly effective drug carriers, benefiting from chitosan’s easy preparation, bioactivity, biodegradability, non-toxicity, biocompatibility, and cationic nature. The versatility of these NPs allows them to integrate both hydrophilic and hydrophobic drugs, expanding their potential uses in medicine. Hydrophilic drugs are blended with CS solution to create a uniform mixture, followed by particle formation through any suitable method. Water-insoluble drugs or those prone to precipitation at acidic pH levels perhaps introduced the formation of post-particles by immersing accomplished nanoparticles in a drug solution at its maximum concentration [90].

**Figure 32:**
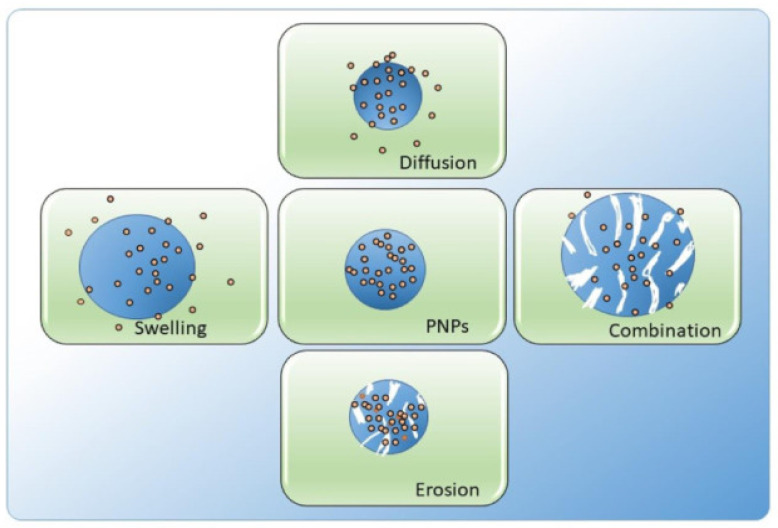
Drug release mechanism from chitosan nanoparticles (CSNPs) [13].

Typically, drug release follows multiple mechanisms. Drug release from CSNPs can occur through four mechanisms: diffusion of adsorbed materials or drug diffusion through the polymeric matrix, swelling of polymers, erosion or degradation of polymers, and a combination of all, as exhibited in Figure 32. For drugs adsorbed on CSNPs’ surfaces or enclosed within the surface layer, the medication rapidly dissolves upon contact with the release medium, causing an initial burst effect. Alternatively, drug release starts slowly due to diffusion and then accelerates [129]. The rate at which drugs are released from their carrier is primarily governed by various formulation and process variables. Drugs’ release from CSNPs is influenced by factors such as particle morphology, size, density, cross-linking degree, chemical and physical characteristics of drugs, and the use of additives. The success of drug delivery systems in a controlled, experimental setting is influenced by several factors, including the pH level (acidity or alkalinity), the polarity of the solution, and the presence of enzymes that may interact with the drug. All these variables are responsible for giving different properties to the final product like matrix density, swelling ability, and degradation abilities [13].

### 4.5. Characteristics of Nanoparticles Used in Drug Delivery Systems

#### Key Morphological Features and Their Impact on the Application of Particles

The size of particles is the critical factor that governs their administration, bio-distribution, bio-accumulation, and elimination from the human body [70]. The oral absorption of particles by M cells or intestinal cells depends on their size, and this size-dependent interaction influences their efficacy and the rate of oral intake. Smaller particles are absorbed by intestinal cells, whereas particles of a larger size are mainly taken up by M cells, filtered by the spleen, and eliminated from the body through kidney excretion [70,130]. Generally, nanoparticles (NPs) with a size exceeding 10 nm tend to evade elimination through renal processes and can infiltrate tissues. Particles within the specific size spectrum of 10–20 nm can translocate through tight endothelial junctions and distribute to various organs. However, they are quickly removed from the bloodstream by the glomeruli. Nanoparticles larger than 200 nm are quickly absorbed by the mononuclear phagocytic system and then accumulate in the spleen and liver. To sum up, in most scenarios, smaller-sized nanoparticles offer the benefit of prolonged circulation and widespread distribution across various tissues [28].

The shape of NPs is important because it affects their efficiency when administrated in vivo. When NPs enter the bloodstream, they can be stopped by a process called phagocytosis, where certain cells try to engulf them. The shape of the NPs makes a big difference in whether this happens. NPs that are not round (like ellipses, bars, or other unusual shapes) can have a strong influence on how they move in our blood vessels and where they end up in our bodies. Even though most NPs studied are spherical, aspherical shapes can cause a big concussion in how they circulate [131].

Hydrophobicity and surface charge are also key factors influencing the behavior of NPs in the body. Combining hydrophilic and hydrophobic materials in NP design can improve their endurance and targeting ability. Surface charge affects NPs’ interaction with cells, potentially affecting their distribution and function in the body [28].

Nanomedicine employs targeting nanocarriers to concentrate particles in specific, disorderly sites. Active targeting, through ligand modification, enhances efficient nanoparticle transport to the desired location, surpassing passive targeting based on properties like size, shape, charge, and hydrophobicity. Tailoring ligands according to disease site features is a future research focus for nanocarrier targeting [95].

### 4.6. Understanding the Mechanism of Intestinal Absorption for CSNPs

The primary obstacle to administering therapeutic molecules orally is the intestinal epithelium, which must be crossed for drugs to enter the systemic circulation. The intestinal epithelium comprises enterocytes, goblet cells, and M cells. Enterocytes, which are the most numerous, facilitate molecule transport through active and passive transport. Goblet cells secrete mucus, acting as a barrier against pathogens. Specialized cells known as M cells, which are located within Peyer’s patches in the intestinal tract, possess the unique ability to take up antigens from the gut and distribute them throughout the body. When therapeutic substances are given orally, they can be absorbed from the intestine via two distinct pathways: transcellular and paracellular routes. CS, a well-known intestinal permeation enhancer, has been shown to facilitate the conveyance of therapeutic molecules through the intestinal epithelial barrier by augmenting both the transcellular and paracellular mechanisms of absorption [29].

#### 4.6.1. Transport Across Cellular Membranes

M cells and enterocytes are crucial in the transcellular transport of CSNPs into the systemic circulation via transcytosis. Adjusting NP characteristics, like size and mucoadhesion, can enhance this process. NPs under 100 nm are easily absorbed by enterocytes, while M cells of Peyer’s patches uptake particles exceeding 500 nm in size [132]. Improving the mucoadhesive characteristics of NPs greatly enhances their ability to traverse epithelial cells. CS, a biopolymer with a positive charge, exhibits strong adhesion to the intestinal mucus layer owing to the electrostatic attraction between the positively charged NPs and the negatively charged sialic acid constituents of the mucus. This potent interaction prolongs the duration of contact between CSNPs and the intestinal lining, thereby promoting enhanced uptake of the NPs within the intestinal tract [133].

#### 4.6.2. Paracellular Transport

Some drugs use the spaces between cells, instead of going through them, to reach their target. This is called paracellular transport. Typically, this movement is hindered by tight junctions (TJs) and the slim gap between adjoining epithelial cells. CS effectively disrupts these TJs, significantly enhancing the paracellular transport of encapsulated molecules. Moreover, the extent of deacetylation and protonation significantly influences CS’s ability to permeate the intestines [134].

## 5. CSNPs as Drug Delivery Systems

CS’s unique biological and chemical properties make it a valuable material for drug delivery systems (DDS) in biomedical and pharmaceutical applications. Its polycationic nature allows it to adhere well to mucous membranes, improving drug contact and penetration. CS is effective for delivering small-molecule drugs and biomolecules like DNA or siRNA. Its transportability is influenced by charge, making it suitable for pH-dependent drug carriers. High deacetylation and molecular weight enhance epithelial permeability, aiding in the transport of polar drugs [35,135]. CS possesses valuable functional properties, but it also comes with certain limitations that impact its applicability in drug delivery. These include limited ability to withstand high temperatures and lacking in flexibility, along with strong affinity to water and notable capacity for expansion through absorption. Its low solubility is another significant feature that restricts its utilization. Enhancing the chitosan’s solubility is a critical factor for its practical and effective application. Chemical modifications of CS offer a means to overcome these limitations and enable its more effective use. CS derivatives are gaining increasing popularity among scientists, leading to the development of more refined and improved modifications. In the pharmaceutical industry, the most prevalent CS derivatives are created through processes such as acylation, carboxymethylation, quaternization, and thiolation [28,70].

CS’s biocompatibility, bioadhesive qualities, and enhanced absorption capabilities make it a valuable biopolymer-based carrier in DDS. CS-based DDS, including nanoparticles, hydrogels, and polymeric hydrogel membranes, are widely employed. Amongst, CSNPs stand out as the most potential candidates for pharmaceutical research. CSNPs share properties with natural and chemically modified polymers and can be prepared under mild conditions, benefiting from chitosan’s solubility under ambient conditions, in aqueous acidic media, circumventing the requirement for hazardous organic solvents or elevated temperatures. CSNPs DDS are versatile systems that can hold various types of drugs, such as small molecules, proteins, and genetic materials. These NPs facilitate the controlled release of the entrapped therapeutic agents. CSNPs can be employed in a wide range of drug delivery routes, including buccal, ocular, oral, nasal, vaginal, pulmonary, periodontal, cutaneous, transdermal modalities, and wound healing. Furthermore, they find utility in the targeted delivery of vaccines and genetic material [28,136]. Studies conducted both in laboratory settings (in vitro) and within living organisms (in vivo) have provided evidence of chitosan’s ability to exhibit anti-tumor properties. This suggests promising potential for its utilization as an adjunctive anticancer drug and as a vehicle for delivering drugs to combat tumors. A study by Pan et al. [137] investigates the promising possibilities offered by CSNPs as a bioadhesive polysaccharide for enhancing the intestinal absorption of insulin. Employing in vivo and in vitro research, the research highlights the potential of CSNPs as a targeted and efficient drug delivery vehicle. By encapsulating insulin, these NPs demonstrate improved bioavailability and absorption within the intestinal tract. This study underscores the significant role of CSNPs in advancing drug delivery technologies, particularly in enhancing the therapeutic efficacy of critical proteins like insulin, and holds promise for improving treatments for various medical conditions.

### 5.1. Enhancing Oral Absorption and Biological Activity of Phytochemical Compounds by Using CSNPs

Phytochemical encapsulation in CSNPs improves their solubility, oral bioavailability, controlled release, and gastrointestinal (GI) stability. Small-sized CS-NPs enhance bioactivity, target specific cells, and reduce extra-organ toxicity. The size of NPs crucially affects their absorption by the intestine’s cells and M cells after administrating by oral route, impacting the rate and extent of absorption. Small NPs are absorbed by intestine’s cells, while large NPs are taken up by M cells [29]. The following paragraphs focus on various phytochemicals and their respective CSNPs, aiming to increase bioactivity and bioavailability across diverse animal models.

#### 5.1.1. Curcumin

Curcumin (CUR), a naturally occurring compound, faces challenges in clinical use due to limited solubility and low bioavailability. Researchers have addressed these issues by encapsulating CUR in CSNPs. In a study conducted by Rahaman et al. [138], curcumin-conjugated chitosan nanomicelles improve CUR’s solubility, stability, sustained release, and oral bioavailability in animal models. Moreover, CUR-loaded complex particles were obtained by ionic cross-linking and polyelectrolyte complexation between CS, gellan, and i-carrageenan [139] and it was demonstrated that the polymer matrix has a protective role of the encapsulated CUR [140]. Overall, CUR-loaded CSNPs offer a promising approach to boosting the efficacy of CUR in clinical use.

#### 5.1.2. Thymoquinone

Thymoquinone (THQ), a potent phytochemical from Nigella sativa, faces challenges in clinical application owing to insufficient solubility and limited bioavailability within biological systems. Scientists created THQ-loaded solid lipid NPs coated with CS (THQ-CS-SLNs) and poly(ε-caprolactone) (PCL)NPs modified with CS (THQ-CS-PCL-NPs). In rat studies, THQ-CS-SLNs exhibited 4.05 times greater intestinal permeation and 3.53 enhanced oral absorption and systemic exposure in comparison to free THQ. Likewise, THQ-CS-PCL-NPs showed 5.34 times increased intestinal permeation and 4.16 times increased oral bioavailability. These results suggest that chitosan-based nanoparticles could be effective in improving the oral delivery of THQ [29].

#### 5.1.3. Ferulic Acid

Ferulic acid (FA), a natural compound in cereals and citrus fruits, has diverse therapeutic potential. However, its clinical application is hindered by low bioavailability. A study by Telange et al. [141] addressed this by developing FA-loaded CSNPs(FA-PLC-CS-NPs). These nanoparticles showed nearly two-fold increased intestinal permeation and notably elevated antioxidant efficacy compared to free FA. Moreover, FA-PLC-CS-NPs exhibited a 2.4-foldincrease in relative oral bioavailability in Wistar rats, indicating their potential to enhance the efficacy of ferulic acid in pharmaceutical uses [29].

#### 5.1.4. Berberine

Berberine (BER), a natural alkaloid compound utilized in treating diarrhea and various illnesses, encounters challenges in clinical usage due to its poor absorption, limited solubility, and lower bioavailability. Nguyen and colleagues [142] developed CS-coated liposomes (BER-CS-L) to enhance oral delivery, resulting in 2.86 and 1.55 times greater oral bioavailability than free BER and uncoated BER-loaded liposomes. Another investigation by Wu et al. [143] employed CS and fucoidan-based NPs to enhance intestinal permeation and maintain the integrity of tight junction barriers, offering the potential for increased oral bioavailability of BER.

#### 5.1.5. Piperine

Piperine (PPN), a natural compound from piper species, shows therapeutic potential but has limited clinical use due to low bioavailability. Zafar et al. [144] devised nanostructured lipid carriers coated with CS loaded with pioglitazone (PPN-CS-NLCs) to improve oral delivery. These carriers displayed robust mucosal adherence, stability within the digestive system, and extended release spanning 24 h. They showcased more than a tenfold increase in intestinal permeation and 3.76 times greater oral bioavailability in rat models when compared to free PPN. Additionally, PPN-CS-NLCs demonstrated enhanced antidiabetic effectiveness in diabetic rats. In conclusion, PPN-CS-NLCs offer a promising nanoplatform for improving PPN’s oral bioavailability and therapeutic effects.

## 6. Limitations and Challenges

In the past few decades, CSNPs have become highly favored due to their exceptional biodegradability, biocompatibility, and non-toxic properties. CSNPs have been shown to substantially increase the intestinal uptake and oral bioavailability of a wide range of phytochemicals. This enhancement is achieved through two primary mechanisms. First, encapsulating phytochemicals within CS-NPs improves their solubility, which is a crucial factor in determining their absorption in the gut. Second, the nanoparticle structure protects the loaded phytochemicals from enzymatic breakdown and pre-systemic metabolism that would otherwise occur in the gastrointestinal tract. By shielding the phytochemicals from these degradative processes, CSNPs enable a greater proportion of the active compounds to reach the bloodstream, thereby boosting their oral bioavailability and potential therapeutic effects [29]. CSNPs face a significant challenge in terms of poor stability, but this issue can be addressed by managing environmental conditions, adjusting temperature, introducing stabilizing compounds, forming a CS-based polymer blend, and modifying the structure of CS using chemical and ionic agents. Another noteworthy disadvantage is the low solubility of CSNPs, limiting their ability to encapsulate hydrophilic drugs, although modifications can enable the encapsulation of water-repellent drugs. The inadequate solubility poses a major obstacle for certain drugs when designing CSNPs. To ensure biocompatibility in humans, toxicology studies and adherence to regulations are crucial. While in vitro studies often yield positive results, these outcomes may not always translate into reality in vivo. Moreover, the commercial viability of a new drug delivery system depends not only on its benefits for patients, but also on its economic feasibility for the pharmacy industries. Scaling up for commercialization is hindered by various experimental factors, such as dialysis and ultracentrifugation [13].

In summary, this review focused on the following most important aspects of chitosan as a nanocarrier in drug delivery and these values are provided in Table 3.

## 7. Future Prospects

Future advancements in controlling drug release from CSNPs are promising. Novel CS nanoparticulate systems, including quaternized CS derivatives, CS cyclodextrin complexes, CS–peptide combinations, thiolated CS, and PEGylated CS, show enhanced properties for oral drug delivery. Identifying optimal systems for encapsulating hydrophilic polyphenols is a key focus for future research, ensuring toxicity verification before progressing into vivo studies [145]. In the near future, research should prioritize commercially viable clinical and pre-clinical studies, focusing on phytochemical-loaded CSNPs. Despite promising results in extensive in vitro and in vivo studies over two decades, no CSNP product for managing chronic diseases has reached the commercial market. To bridge this gap, it is imperative to conduct clinical trials, ensuring economic viability. Moreover, it is essential to employ simple and scalable preparation methods for the mass production of CSNPs, ensuring that these techniques fall within the “Generally Recognized as Safe” (GRAS) category, as many academic methods are not licensed for commercial applications. Once preparation methods are established, thorough investigations involving animals and humans are needed to assess bioavailability, safety, and bioactivity following oral administration. Ongoing research on diverse CS sources, doses, administration routes, and deacetylation degrees is crucial for understanding their effective utilization in animal production, presenting significant potential for enhancing animal health and productivity [146].

## 8. Conclusions

This review mainly discussed the most interesting papers published in the last ten years. In conclusion, the versatile applications of CS-based nanomaterials, particularly CSNPs with sizes ranging from 100 to 400 nm in various biomedical domains, underscore their immense potential to advance drug delivery systems. The distinctive characteristics of CS, such as biocompatibility, biodegradability, and abundant functional groups, make it a highly appealing option for applications involving controlled release. Chemical modifications, including sulfation, thiolation, and cycloaddition, further enhance the biological activities of CS, expanding its utility in nanotechnological fields, such as wound dressing, cancer therapy, gene delivery, and biosensors. Despite the promising prospects, challenges remain, especially in ensuring the safety and bioactivity of CS-based nanocarriers in vivo, necessitating comprehensive evaluations for future commercialization. The dialysis technique is frequently utilized to evaluate drug release from CSNPs, unveiling a two-phase pattern characterized by an initial rapid release followed by a controlled and pH-dependent release. The review emphasizes the pivotal role of CSNPs in functional drug delivery systems, offering improved oral bioavailability and bioactivity, with modifications in CS structure, enhancing mucoadhesive characteristics and controlled release. Additionally, CSNPs, with their substantial surface area, present a significant advantage in biomedical applications. Ongoing research endeavors focus on refining CS properties through chemical modifications and exploring innovative nanoparticulate formulations for safe, biocompatible, and target-specific drug delivery. Although challenges persist, systematic studies on biodistribution, toxicity, and selectivity are imperative for the continued development and optimization of CS-based nanocarriers, holding promise for the future of effective and tailored drug delivery systems in biomedical, pharmaceutical, and food industries.

## Figures and Tables

**Table 1:** The purity of chitin obtained from different raw materials and by different methods.

Marine Habitat	Chitin Source	CaCO_3_ (%)	Protein (%)	Other (%)	Purity (%)	Yield (%)
Chemical Method						
All seas except polar	Lobster shells (*Nephropidae*)	0.39 ± 0.23	2.22 ± 0.24	3.93 ± 0.09	93	17.21 ± 0.28
All seas except polar	Lobster shells (*Nephropidae*)	0.30 ± 0.20	2.90 ± 0.25	4.17 ± 0.03	93	16.53 ± 2.35
Indian and North Pacific Ocean	Shrimp shells (*Marsupenaeus japonicus*)	0.45 ± 0.10	1.13 ± 0.01	1.32 ± 0.00	97	16.08 ± 0.57
DES—mediated method						
Coastal mud shrimp	Shrimp shells (*Solenocera crassicornis*)	0.3–0.4	0.5–0.6	-	99.1 ± 0.1	4.9v1
Coastal mud shrimp	Shrimp shells (*Solenocera crassicornis*)	0.6–0.7	07–0.8	-	98.6 ± 0.2	13.2 ± 1.1
Indian and North Pacific Ocean	Japanese tiger prawn (*Marsupenaeus japonicus*)	0.74	0.74 ± 0.02	1.53 ± 0.02	23.86 ± 0.07	3.72 ± 0.05
North Atlantic Ocean	Shrimp shells (*Solenocera crassicornis*)	0.56	0.98	0.46	98 ± 1	19–20
All seas except polar	Lobster shells (*Nephropidae*)	0.21 ± 0.31	1.81 ± 0.14	4.12 ± 0.21	93	22.21 ± 0.27
All seas except polar	Lobster shells (*Nephropidae*)	0.34 ± 0.22	1.77 ± 0.22	3.88 ± 0.11	93	21.01 ± 0.23
All seas except polar	Lobster shells (*Nephropidae*)	0.24 ± 0.16	1.75 ± 0.17	4.11 ± 0.23	93	19.01 ± 0.24
Indian and North Pacific Ocean	Japanese tiger prawn (*Marsupenaeus japonicus*)	1.44 ± 0.01	3.59 ± 0.02	1.92 ± 0.01	93	25.00 ± 0.60
Indian and North Pacific Ocean	Japanese tiger prawn (*Marsupenaeus japonicus*)	1.18 ± 0.01	8.37 ± 0.05	2.32 ± 0.06	88	25.18v0.38
Indian and North Pacific Ocean	Japanese tiger prawn (*Marsupenaeus japonicus*)	3.60 ± 0.14	13.05 ± 0.20	1.25 ± 0.04	82	25.22 ± 0.90
Indian and North Pacific Ocean	Japanese tiger prawn (*Marsupenaeus japonicus*)	41.01 ± 1.80	15.34 ± 0.18	4.56 ± 0.02	39	50.54 ± 1.07
Indian and North Pacific Ocean	Japanese tiger prawn (*Marsupenaeus japonicus*)	44.34 ± 3.40	13.50 ± 0.12	4.42 ± 0.04	38	52.45 ± 2.01
Indian and North Pacific Ocean	Japanese tiger prawn (*Marsupenaeus japonicus*)	46.19 ± 1.90	16.14 ± 0.10	6.42 ± 0.09	31	52.55 ± 0.70

**Table 2:** Different sizes and types of chitosan.

Types of Chitosan Biopolymer	Properties	
	Physicochemical Properties	Biological Properties
Low MW Chitosan (LMWC < 150 kDa)	LMWC possesses a higher solubility, permeability, and tensile strength, but lower viscosity than other melting points with reduced thermostability. It is widely used in food, agriculture, medical, and pharmaceutical fields.	Comparatively, LMWC is more bioactive than other types of chitosan due to the presence of short low-mw chains. Also, the higher degree of deacetylation is associated with increased bioactivity. As per studies, the relationship between chitosan’s mw and cytotoxicity is controversial.
Medium MW Chitosan (MMWC = 150–750 kDa)	The aqueous solubility of MMWC is less then LMWC but more than HMWC. Permeability and viscosity are in between the range of HMWC and LMWC, and they exhibit high tensile strength.	They have the best antioxidant properties among the three. The potential of MMWC film has been confirmed by several studies.
High MW Chitosan (HMWC > 750 kDa)	Viscosity of HMWC is high but solubility, permeability, and tensile strength are lower.	It can be used as an antibacterial coating in the food industry. It shows the least amount of antioxidant properties due to the presence of more intermolecular hydrogen bonds in the longer chain, resulting in less accessibility to the free radicals.

**Table 3:** Important values concerning the chitosan.

Important Values	Description
Sources and structure of CS:	CS is a mucopolysaccharide that occurs naturally, which resembles cellulose by chemical structure but differs in having a functional group of acetylamino. Depending on the origin of the chitin, its physicochemical properties are notably controlled by both its molecular weight and degree of deacetylation, including its solubility in various solvents and pH levels, hydrophobicity, and toxicity.
Basic characteristics of CS:	The biocompatible, non-toxic, mucoadhesive, and biodegradable chitosan biopolymer is soluble in an acidic aqueous solution, and its solubility is influenced by its molecular weight and degree of deacetylation.
Drug delivery properties of CS:	The positively charged characteristic of CS is likely responsible for its mucoadhesive characteristics. Chitosan can be used to deliver the therapeutics to the body in the form of film, hydrogel, composites, nanoparticles, nanocomposite, scaffolds, nanocarriers etc.
Enhancing CS’s properties through chemical modifications:	Chitosan molecules can be functionalized into different derivatives for desired and improved physicochemical and biological properties.
Preparation methods of CS-based nanoparticles:	Chitosan nanoparticles can be prepared by using different methods like ionic gelation, solvent diffusion, solvent evaporation methods, nanoprecipitation, the desolvation method, and so on.
Administration routes for CSNPs:	Chitosan nanoparticles can be delivered via many routes like ocular, oral, transdermal etc. They are also used for active targeting and vaccine delivery.
Enhancing oral absorption and biological activity of phytochemical compounds by using CSNPs:	Phytochemical encapsulation in CSNPs improves their solubility, oral bioavailability, controlled release, and gastrointestinal (GI) stability. Small-sized CS-NPs enhance bioactivity, target specific cells, and reduce extra-organ toxicity.

## Data Availability

No new data were created or analyzed in this study. Data sharing is not applicable to this article.
